# Pediatric Liver and Transplant Surgery: Results of an International Survey and Expert Consensus Recommendations

**DOI:** 10.3390/jcm12093229

**Published:** 2023-04-30

**Authors:** Caroline P. Lemoine, Omid Madadi-Sanjani, Claus Petersen, Christophe Chardot, Jean de Ville de Goyet, Riccardo Superina

**Affiliations:** 1 Division of Transplant and Advanced Hepatobiliary Surgery, Ann & Robert H. Lurie Children’s Hospital of Chicago, Northwestern University Feinberg School of Medicine, Chicago, IL 60611, USA; 2 Department of Pediatric Surgery, Hannover Medical School, 30625 Hannover, Germany; 3 Service de Chirurgie Pédiatrique Viscérale, Hôpital Necker—Enfants Malades, Université de Paris, 75015 Paris, France; 4 Department for the Treatment and Study of Pediatric Abdominal Diseases and Abdominal Transplantation, ISMETT, 90127 Palermo, Italy

**Keywords:** pediatric liver surgery, pediatric liver transplantation, hepatoblastoma, hepatocellular carcinoma, pediatric surgery workforce, subspecialization

## Abstract

Background: Pediatric liver surgery is a complex and challenging procedure and can be associated with major complications, including mortality. Best practices are not established. The aims of this study were to evaluate surgeons’ individual and institutional practices in pediatric liver surgery and make recommendations applicable to the management of children who require liver surgery. Methods: A web-based survey was developed, focusing on the surgical management of children with liver conditions. It was distributed to 34 pediatric surgery faculty members of the Biliary Atresia and Related Disorders (BARD) consortium and 28 centers of the European Reference Network—Rare Liver. Using the Delphi method, a series of questions was then created to develop ideas about potential future developments in pediatric liver surgery. Results: The overall survey response rate was 70.6% (24/34), while the response rate for the Delphi questionnaire was 26.5% (9/34). In centers performing pediatric liver surgery, most pediatric subspecialties were present, although pediatric oncology was the least present (79.2%). Nearly all participants surveyed agreed that basic and advanced imaging modalities (including ERCP) should be available in those centers. Most pediatric liver surgeries were performed by pediatric surgeons (69.6%). A majority of participants agreed that centers treating pediatric liver tumors should include a pediatric transplant program (86%) able to perform technical variant grafts and living donor liver transplantation. Fifty-six percent of responders believe pediatric liver transplantation should be performed by specialized pediatric surgeons. Conclusion: Pediatric liver surgery should be performed by specialized pediatric surgeons and should be centralized in regional centers of excellence where all pediatric subspecialists are present. Pediatric hepatobiliary and transplant training needs to be better promoted amongst pediatric surgery fellows to increase this subspecialized workforce.

## 1. Introduction

Hepatoblastoma and hepatocellular carcinoma (HCC) are the two most common primary liver tumors affecting children and teenagers. However, despite this, those tumors constitute rare diseases. It is estimated that approximately 100 children are treated for hepatoblastoma at nearly 100 different institutions on an annual basis in the United States [1,2]. Therefore, the number of liver resections performed at each individual center and by individual pediatric surgeons is extremely low, and the management is not uniform. Pediatric liver surgery can also be performed for benign tumors or infectious conditions. 

Over the last 33 years in the United States, the number of pediatric surgery training programs has increased by 278%, and the number of pediatric surgeons increased by 132% [3]. The consequence of this increase in the workforce has been a reduction in index cases per individual surgeon. On average, each U.S. surgeon performed less than one Kasai portoenterostomy or choledochal cyst excision per year. Liver resection was not evaluated in that study. 

Studies from both the adult and pediatric surgery literature have shown improved outcomes with the increased surgeon and center volume for rare conditions [4,5]. In children, a relationship between volume and outcomes has been shown in Kasai portoenterostomy [6], congenital diaphragmatic hernia repair [7], and Wilms tumor resection [8]. The success of pediatric liver surgery is obviously not solely related to the surgeon performing the surgery but also to the presence of other pediatric specialists and appropriate resources at an institution to optimally diagnose, treat, and care for these children with complex conditions. 

In this study, an international group of pediatric hepatobiliary and transplant surgeons aimed to evaluate and compare their individual and institutional practice in pediatric liver surgery and to elaborate expert recommendations in the management of children with liver tumors. 

## 2. Methods 

### 2.1. Development of the Questionnaire

A panel of experts consisting of pediatric hepatobiliary surgeons was created. Based on author consensus, a total of 31 questions were generated, focusing on different aspects of the management of pediatric liver conditions requiring either liver resection or transplantation: (1) institutional logistics related to the management of children with liver disease requiring surgical intervention; (2) surgical management, including indication for surgery, surgical technique, duration of surgical interventions and estimated blood loss, postoperative hospital stay; (3) oncology management for malignant conditions; (4) pediatric liver transplantation. The detailed content of the questionnaire is available in Supplementary Materials Document S1. 

### 2.2. Study Design

The web-based questionnaire was developed in English. The questionnaire was a self-administered, web-based survey using the online tool SurveyMonkey (http://www.surveymonkey.com, accessed on 11 August 2020). Each participant could advance in the survey after skipping a question. There were no mandatory questions. 

### 2.3. Study Population

The questionnaire was electronically distributed to 34 faculty members of the Biliary Atresia and Related Disorders (BARD) consortium and 28 centers of the European Reference Network—Rare Liver. The questionnaire did not differentiate between free-standing pediatric hospitals or pediatric departments part of an adult healthcare institution. 

The survey was answered by a variety of surgeons: pediatric surgeons or transplant surgeons (either pediatric or adult surgeons performing pediatric liver transplant or pediatric hepatobiliary surgery) located in Europe, North America, Asia, and Australia. 

### 2.4. Distribution of the Questionnaire 

The survey was distributed by email. A cover letter clearly detailed the objectives of the survey. Survey administration followed the Dillman principles and recommendations of Burn et al. [9]. Participants were first contacted on 11 August 2020. A total of 2 reminder emails were sent 2 and 4 weeks later. The survey was closed on 9 December 2020. Only one participant was allowed to answer in each individual institution. Overall, 24 pediatric surgeons answered the survey. 

### 2.5. Development of the Delphi Questionnaire 

The variables assessed in the Delphi questionnaire were selected by consulting an international panel of experts in pediatric hepatobiliary surgery. The questionnaire was developed using a semi-structured interview with the aim of identifying redundant or poorly worded questions. Testing of the questionnaire was performed by running the questions to 10 other pediatric surgeons and hepatologists. Last, the reliability of the questionnaire was assessed by re-testing the same pediatric surgeons with the questionnaire at a 2-week interval. Participants were allowed to answer the Delphi questionnaire between 5 March 2021 and 29 April 2021. Nine surgeons answered the Delphi questionnaire (26.5% response rate, 9/34). 

### 2.6. Pre-Meeting Working Group 

A working group was created constituted of four surgeons (CL, OMS, RS, and CP). They analyzed the results of the Delphi questionnaire and organized a summary presentation of the survey and Delphi questionnaire results. 

### 2.7. Expert Panel Meeting 

The results of the survey and the Delphi questionnaire were presented and discussed within an expert panel meeting during the virtual BARD webinar held on 30 June 2021. Other participants in the webinar could also participate through online chat. Panelists were provided with a summary depicting the results of the survey and the Delphi questionnaire. RS served as moderator of the meeting session. 

## 3. Results 

### 3.1. Institutional Logistics Related to the Pediatric Liver Surgery 

The survey was answered by 24 surgeons. The annual number of pediatric liver surgical interventions performed in each institution varied greatly from 2 to >100. Pediatric surgeons were present in 100% of institutions. The transplant surgery was present in 87.5% (21/24) of cases. Pediatric anesthesiology (95.8%), pediatric hepatology, and pediatric radiology (91.7%) were also often present. Pediatric oncology was the pediatric specialty that was the least present at those institutions (79.2%). A pediatric intensive care unit was present in all hospitals, while a pediatric ward was nearly always present (95.8%, 23/24). The number of beds dedicated to pediatric patients ranged from 10 to 500, while the number of beds for pediatric surgery patients ranged from 10 to 250. 

General imaging modalities (computed tomography (CT) or magnetic resonance imaging (MRI)) were available in all centers. However, interventional radiology (IR) with the ability to perform advanced procedures (percutaneous transhepatic cholangiogram or portal vein embolization) was available in 91.7% (22/24) of institutions. Diagnostic endoscopic retrograde cholangiopancreatography (ERCP) was available in 87.5% of cases, but interventional gastroenterologists able to perform bile duct stenting in small infants were present in 66.7% of surveyed institutions (16/24). 

When surveyed through the Delphi questionnaire, all participants agreed that centers performing pediatric liver surgery should provide ultrasound, CT, MRI, and diagnostic interventional radiology. Almost all agreed advanced IR interventions (98%) and ERCP (91%) should also be available. 

### 3.2. Surgical Management 

All surveyed participants reported that liver surgery for benign and malignant tumors was performed at their institution. Hepatoblastoma and HCC were the two most common indications. Trauma surgery (73.9%, 17/24) and surgery for infectious causes (abscess, hydatic cysts) (69.6%, 16/24) were performed less frequently. 

**Figure 1:**
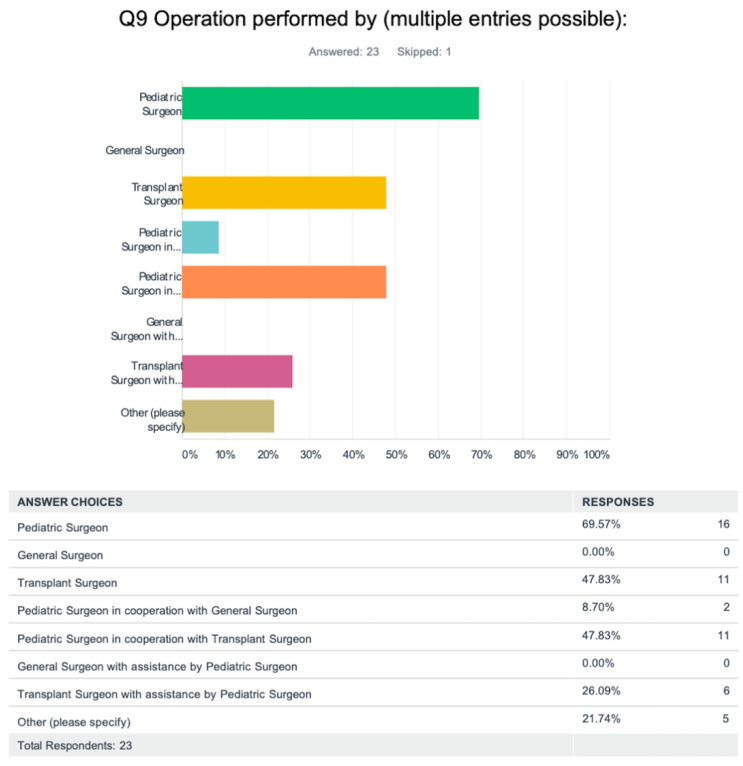
Questionnaire asking which provider performs pediatric liver surgery at surveyed centers. (Each color represents a different answer).

Most pediatric liver surgeries were performed by a pediatric surgeon (69.6%). (Figure 1) Less frequently, a transplant surgeon (47.8%) or a pediatric surgeon assisted by a transplant surgeon (47.8%) performed the intervention. In no instance did an adult general surgeon perform liver surgery on children. Of note, the survey did not specifically evaluate pediatric surgeons with additional training in hepatobiliary and/or transplant surgery, nor did it identify dedicated transplant surgeons who perform only pediatric transplants. 

**Figure 2:**
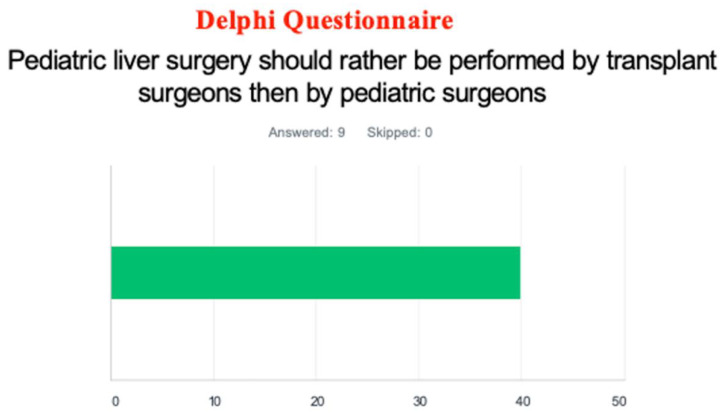
Questionnaire asking who should perform pediatric liver surgery: pediatric vs. transplant surgeons. (The color represents the proportion of answers provided).

Through the Delphi questionnaire, most surveyed participants (60%) answered that pediatric liver surgery should be performed by a pediatric surgeon rather than a transplant surgeon. (Figure 2) A majority of responders (64%) recommended that a pediatric liver tumor surgical team include a pediatric surgeon and a transplant surgeon. 

All responders reported being able to perform pediatric liver surgery via laparotomy (most frequently through a transverse laparotomy with a midline extension; 40.9%), while only 47.8% reported also using a laparoscopic approach. The Delphi questionnaire revealed that the participants were evenly divided regarding which surgical approach should be used for pediatric liver surgery and if laparoscopic constitutes a valuable option for both minor and major pediatric liver surgical interventions (50%). 

Standard left/right hepatectomy was performed at all institutions surveyed, while non-anatomical liver resection and extended left/right hepatectomy were performed in a majority of centers (22/24, 95.7%). The average operation duration ranged from 60 to 240 min for all liver resections except for the extended right (150–300 min) or left (150–360 min) hepatectomy. Estimated blood losses were lower for standard left/right hepatectomy or non-anatomical resection (20–500 mL) compared to extended hepatectomies (25–750 mL). The utilization of total vascular exclusion of the liver and inflow exclusion (Pringle maneuver) were utilized as needed. Five responders (22.7%) reported not utilizing any type of vascular exclusion for their liver resections, although the question did not specify in which instance vascular exclusion would or could be used. Most participants (72.7%, 16/24) reported using the Cavitron ultrasonic surgical aspirator (CUSA) for the parenchymal dissection. The non-stick bipolar diathermy (59.1%) and the “clamp crush” technique (50.0%) were other commonly used techniques. Most participants reported placing a surgical drain at the completion of liver resection (95.7%, 22/24). Overall hospital length of stay varied from 2 to 30 days, and intensive care unit stay ranged from 1 to 10 days. 

The Delphi questionnaire showed that while most participants believe the knowledge of different vascular exclusion techniques is mandatory to perform pediatric liver surgery (83%), less than a third believe a Pringle maneuver is necessary during parenchymal dissection, and forty-two percent believe total vascular exclusion should be prepared for major liver resections. The majority (94%) thought the CUSA or other such equipment should be available in centers performing pediatric liver surgery. 

### 3.3. Oncology Management 

All surgeons answered that the oncological management of their pediatric patients with liver tumors is performed by a pediatric oncologist. However, the chemotherapy administration is performed at the same center in only two-thirds of cases. In the other third, patients are treated at another center. Most institutions (22/24, 95.7%) decide on postoperative oncological management at interdisciplinary oncology boards. 

### 3.4. Pediatric Liver Transplantation 

Pediatric liver transplantation was performed in most centers surveyed (20/24, 87.0%). In most instances, either a pediatric surgeon (57.1%) and/or a transplant surgeon (61.9%) performed the transplant. The post-transplant management was mostly performed by pediatric hepatologists (90.5%) and assisted by pediatric or transplant surgeons (52.4%). The survey did not specify if this question pertained to the short- or long-term management of transplant recipients. 

**Figure 3:**
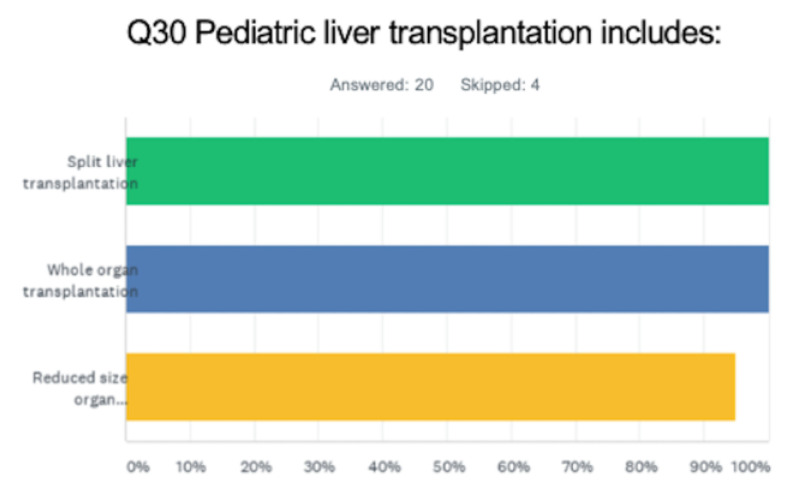
Different types of deceased donor liver transplantation graft types offered at surveyed centers. (Each color represents a different answer).

All centers performed deceased donor liver transplants, while most also performed living donor liver transplants (95%, 19/20). Only 25% of centers accepted organs from donors after cardiac death. All participants responded that split liver transplantation was performed at their institution in addition to whole liver transplantation. (Figure 3) Most programs offered multi-organ transplants (simultaneous liver–kidney transplant: 17/18, 94.4%; less frequently intestinal or multivisceral transplant: 7/18, 38.9%). 

**Figure 4:**
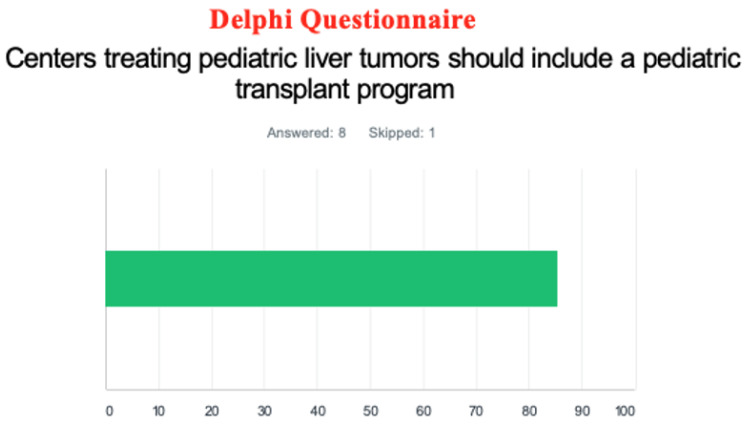
Questionnaire asking if centers treating pediatric liver tumors should include a pediatric transplant program. (The color represents the proportion of answers provided).

**Figure 5:**
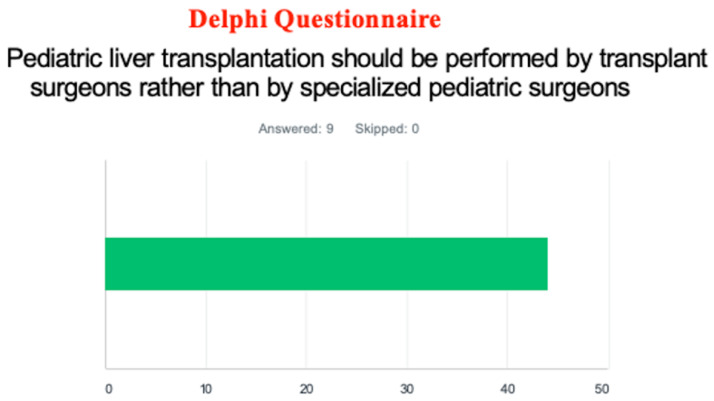
Questionnaire asking who should perform pediatric liver transplantation: pediatric vs. transplant surgeons. (The color represents the proportion of answers provided).

The Delphi questionnaire showed most responders believed centers treating pediatric liver tumors should include a pediatric transplant program (86%). (Figure 4) Although the distribution was almost equal, a slight majority of responders believed pediatric liver transplantation should be performed by specialized pediatric surgeons (56%). (Figure 5) 

## 4. Discussion

Pediatric hepatobiliary surgery constitutes a challenging surgical intervention, with reported complication rates between 10–30% and a mortality rate of 5% [10,11,12,13,14]. In order to achieve the best outcomes for children with surgical liver conditions, a multidisciplinary team of pediatric specialists is needed. As presented by the World Federation of Associations of Pediatric Surgeons (WOFAPS) Declaration of Pediatric Surgery in 2001: “To provide the best surgical care for infants and children, complex pediatric surgical procedures should be carried out in specialized pediatric centers with appropriately equipped intensive care facilities staffed 24 h per day, 7 days per week. In addition to the trained pediatric surgeons, these facilities should be staffed with other pediatric specialists including radiologists, anesthesiologists, and pathologists” [15]. 

As previously presented, complex hepatobiliary surgeries are now less frequently performed by general pediatric surgeons. In the study by Abdullah et al., 60% of surveyed pediatric surgeons had not performed either a Kasai portoenterostomy or a choledochal cyst excision in the previous 12 months [3]. Liver resection is so rarely performed that it was not even compiled in the list of procedures evaluated in that study. A 2016 study focused on subspecialization in North American pediatric surgery groups showed that major liver resection (left/right hepatectomy or extended resection) was performed by any surgeon of a group in approximately 45% of cases and was performed by only certain dedicated surgeons in less than 10% [16]. Recently, a group from Texas reported an improvement in postoperative complications after major liver resection (52% to 20%) when they limited pediatric liver resections to be performed by only 2 surgeons rather than anyone in a group of 10 surgeons (previously creating an average of 3.3 cases/surgeon over a 10-year period) [2]. Limiting the number of surgeons performing liver resections in a group allows those hepatobiliary surgeons to increase their experience, becoming capable of performing all types of liver resections and offering the best outcomes to patients. 

Subspecialization in hepatobiliary surgery is not yet recognized by most general pediatric surgeons. The most common specialties felt to be necessary are transplantation, fetal interventions, and bariatric surgery [17]. Most respondents of an American Pediatric Surgery Association (APSA) survey felt that specialists should not practice solely in their subspecialty but rather act as content experts and consult on relevant cases. Why pediatric surgeons are so reluctant to accept the benefits of subspecialization and concentrating cases on a subgroup of surgeons is multifactorial: it might not be easily applicable in rural settings or small groups/practices [16,18,19]. Additional years of formal training (hepatobiliary and/or transplant fellowship) can be difficult to achieve for personal or financial reasons. Nevertheless, in that same APSA survey, 50.8% of respondents responded that additional specialization training is necessary after completing a fellowship in general pediatric surgery. 

Unlike adult general surgery, general pediatric surgery has been hanging on to the concept of the true general surgeon, even in academic centers. A 2010 survey of general surgery residency program directors reported that 71% of finishing general surgery residents entered a subspecialty residency [20]. Pediatric surgery subspecialty fellowships (fetal, colorectal, vascular anomalies, hepatobiliary, and trauma) are becoming more available, but the true translation of this concept into the reality of daily practice remains unfulfilled, particularly in the field of hepatobiliary surgery. 

Hepatobiliary and pancreatic surgery deals with some of the most complicated and technically challenging operations. A survey of Canadian general surgeons showed that 91% of respondents would refer patients with the complicated hepatobiliary disease to a hepatobiliary expert [21]. Additionally, 95% of participants thought that some hepatobiliary procedures should be regionalized to high-volume, expert centers: pancreaticoduodenectomy, biliary reconstruction, and major hepatectomy (defined as two or more liver segments). In adult surgery, a relationship between outcomes and high-volume centers has been demonstrated. 

Additionally, training in transplant surgery allows pediatric surgeons to offer all surgical treatment options to patients referred for the management of malignant liver tumors. Lautz et al. showed that of 18 patients, who were referred for liver transplantation by other pediatric surgeons and institutions because of what appeared to be unresectable tumors, all were resected, and their survival was equal or superior to that of patients treated with liver transplantation [22]. 

In the survey presented in this study, living donor liver transplantation was performed in 95% of centers and split liver transplantation in 100% of programs. This proportion is very different from most programs performing pediatric liver transplantation in the U.S. However, the authors believe that any pediatric liver transplant program should have the ability to offer all types of transplants, including technical variant grafts and living donor transplantation, in order to decrease the waitlist mortality as much as possible. 

As reported in our international survey, some institutions rely on an adult transplant surgeon collaborating with a pediatric surgeon on liver resection in children and teenagers [19]. While the authors agree that the safety of the patient is the most important consideration in pediatric liver surgery and that a pediatric surgeon uncomfortable with performing a liver surgery should not attempt such an endeavor on their own, relying on an adult surgeon should not be the default solution. Again quoting Dr. Grosfeld in the WOFAPS Declaration of Pediatric Surgery, “Children are not just small adults and have medical and surgical problems and needs that often are quite different from those encountered by adult physicians. [15] Every infant and child who suffers from an illness or disease has the right to be treated [15] by a pediatric medical or surgical specialist” [15]. The American Academy of Pediatrics (AAP) also issued a policy statement regarding referral to pediatric surgical specialists [23]. The Surgical Advisory Panel recommended that “infants, children, and adolescents with solid malignancies should be cared for from the outset by a pediatric surgeon or a pediatric surgical specialist and a pediatric medical cancer specialist.” 

In conclusion, the authors and the participants of the BARD webinar propose the following recommendations for the management of pediatric patients with liver disorders requiring surgical treatment:

1. Pediatric liver surgery should be performed in institutions where all pediatric medical and surgical specialists are available on site to provide specialized pediatric care;
2. Pediatric liver surgery should be performed in centers where advanced interventional radiology and interventional gastroenterology are available to help provide all possible diagnostic and therapeutic adjuncts procedures to the management of children with liver conditions;
3. Pediatric liver surgery should be performed in centers where pediatric liver transplantation is performed (including technical variant grafts and living donor liver transplantation) and where surgical oncologists are present in order to offer the best oncological management;
4. Pediatric liver surgery should be performed by pediatric surgeons subspecialized in hepatobiliary and transplant surgery to allow knowledge and mastering of all surgical techniques, including vascular exclusion techniques;
5. Pediatric liver surgery should be centralized in regional centers of excellence in pediatric hepatobiliary surgery;
6. Subspecialization in hepatobiliary and transplant surgery should be promoted amongst pediatric surgery trainees; additionally, incentives or compensation strategies should be developed to help support pediatric surgeons through additional years of training and to avoid trainees being reluctant to complete subspecialty training.

## Data Availability

No new unavailable data were created, all datasets analyzed or generated during the study are included in this manuscript or in the supplemental material provided by the authors.

## Supplementary Materials

The following supporting information can be downloaded at: https://www.mdpi.com/article/10.3390/jcm12093229/s1, Figure S1: Liver Surgery Survey and Delphi questionnaire.

## Abbreviations

CT	Computed tomography
CUSA	Cavitron ultrasonic surgical aspirator
ERCP	Endoscopic retrograde cholangiopancreatography
HCC	Hepatocellular carcinoma
IR	Interventional radiology
MRI	Magnetic resonance imaging
PTC	Percutaneous transhepatic cholangiogram
