# Supplementary material for: Pediatric Liver and Transplant Surgery: Results of an International Survey and Expert Consensus Recommendations
Source: J Clin Med. 2023 Apr 30;12(9):3229. doi: 10.3390/jcm12093229 (PMC10179485; doi:10.3390/jcm12093229)
Supplement: Supplementary file 1 [file jcm-12-03229-s001.zip › jcm-2287053-supplementary.pdf]

Current concepts of pediatric liver surgery and pediatric liver transplantation

Tuesday, June 29, 2021

24

Total Responses

Date Created: Tuesday, August 11, 2020

Complete Responses: 24

Q2: Hospital departments at your Institution:

Answered: 24   Skipped: 0

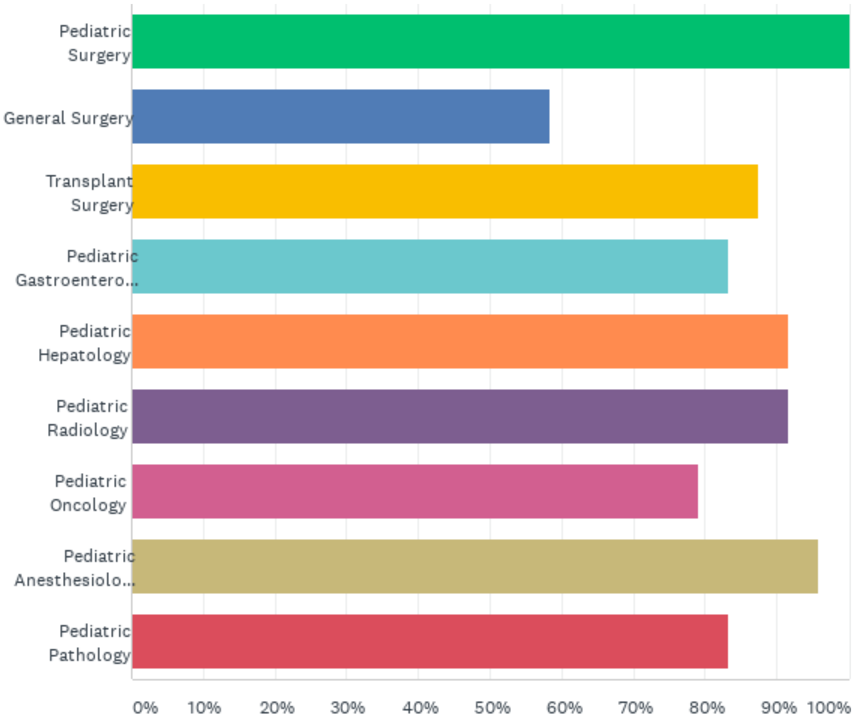

Powered by SurveyMonkey

Q2: Hospital departments at your Institution:

Answered: 24   Skipped: 0

ANSWER CHOICES	RESPONSES	
Pediatric Surgery	100.00%	24
General Surgery	58.33%	14
Transplant Surgery	87.50%	21
Pediatric Gastroenterology	83.33%	20
Pediatric Hepatology	91.67%	22
Pediatric Radiology	91.67%	22
Pediatric Oncology	79.17%	19
Pediatric Anesthesiologist	95.83%	23
Pediatric Pathology	83.33%	20
Total Respondents: 24		

Powered by SurveyMonkey

Q3: Logistics at your Institution:

Answered: 24 Skipped: 0

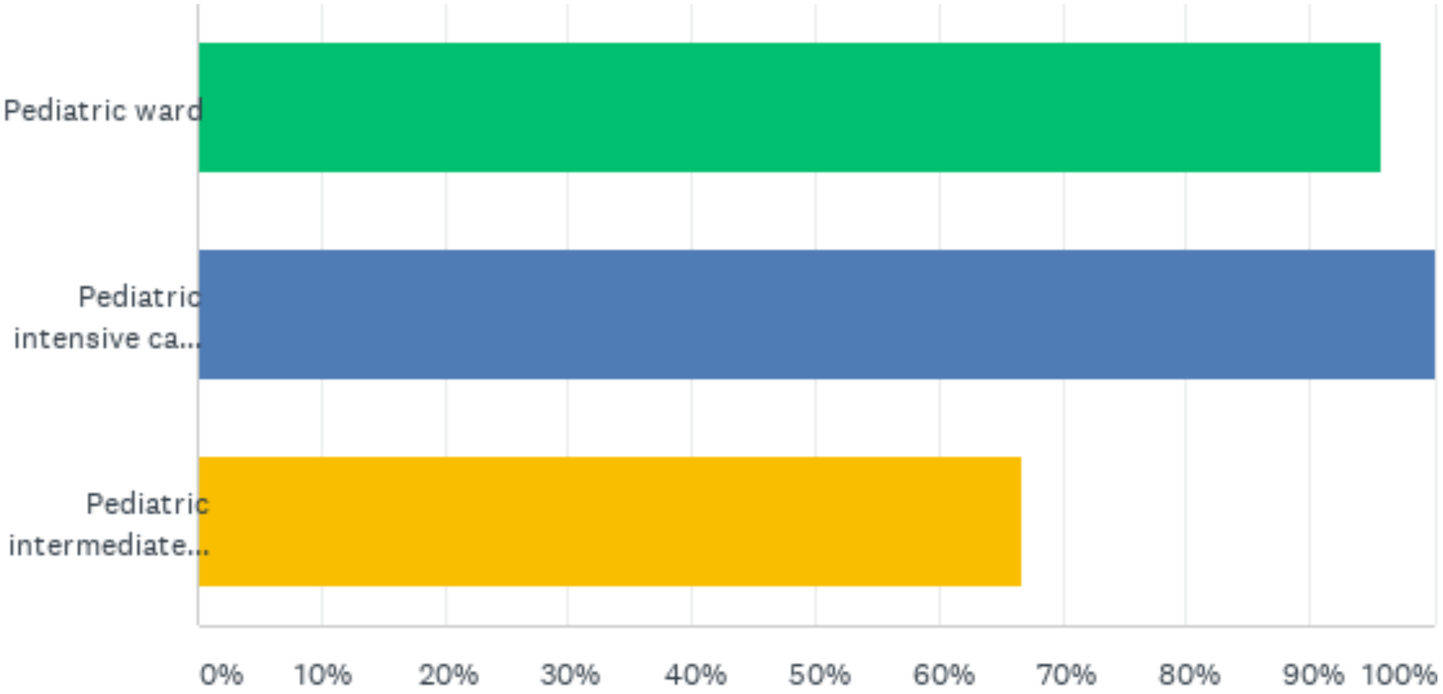

Powered by SurveyMonkey

Q3: Logistics at your Institution:

Answered: 24   Skipped: 0

ANSWER CHOICES	RESPONSES	
Pediatric ward	95.83%	23
Pediatric intensive care unit	100.00%	24
Pediatric intermediate care unit	66.67%	16
Total Respondents: 24		

Powered by SurveyMonkey

Q6: Imaging facilities for children at your Institution:

Answered: 24   Skipped: 0

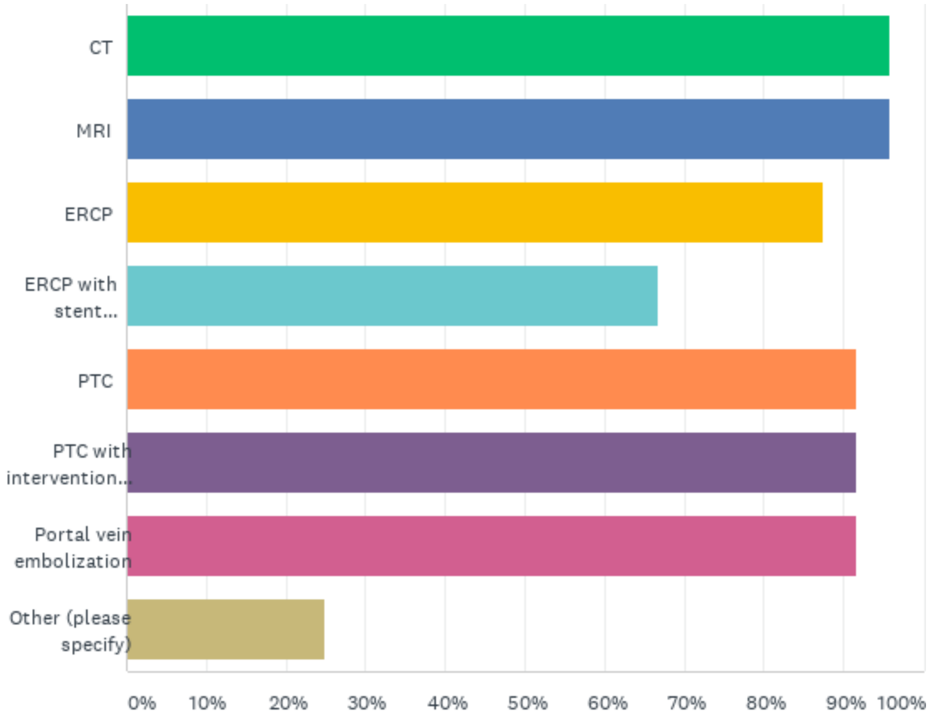

Q6: Imaging facilities for children at your Institution:

Answered: 24 Skipped: 0

ANSWER CHOICES	RESPONSES	
CT	95.83%	23
MRI	95.83%	23
ERCP	87.50%	21
ERCP with stent Implantation in infants	66.67%	16
PTC	91.67%	22
PTC with interventions (e.g. Dilatation, stenting)	91.67%	22
Portal vein embolization	91.67%	22
Other (please specify)	25.00%	6
Total Respondents: 24		

Q7: Reason for liver resection:

Answered: 23   Skipped: 1

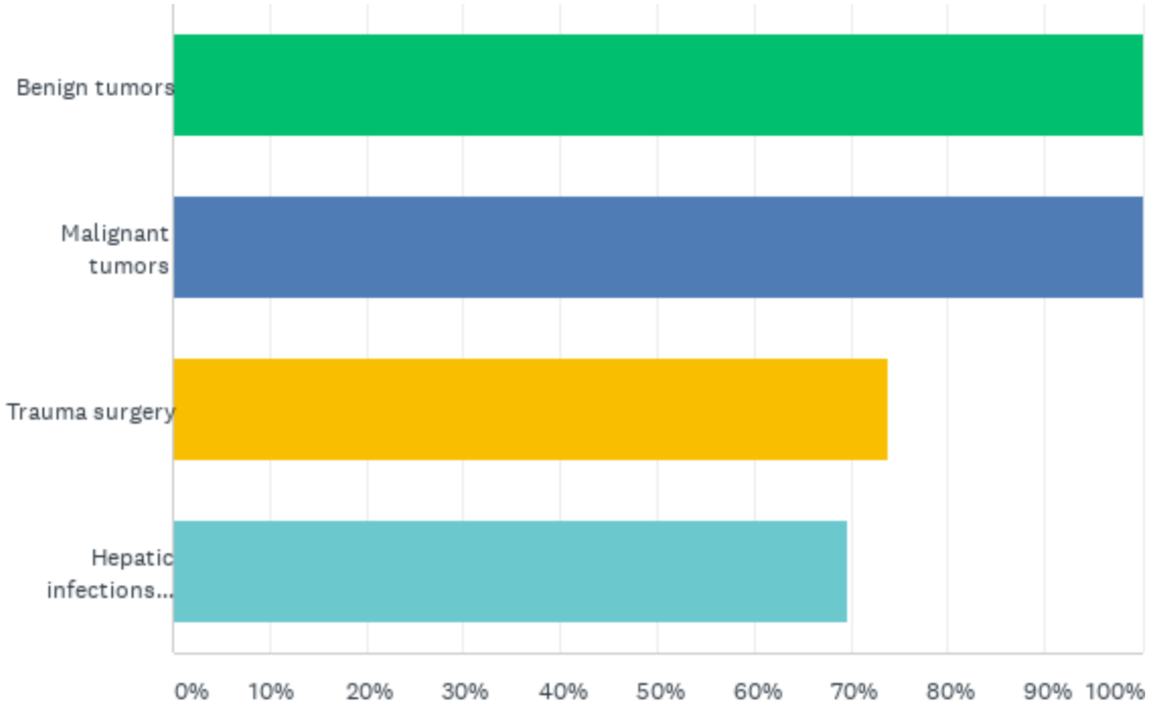

Powered by SurveyMonkey

Q7: Reason for liver resection:

Answered: 23   Skipped: 1

ANSWER CHOICES	RESPONSES	
Benign tumors	100.00%	23
Malignant tumors	100.00%	23
Trauma surgery	73.91%	17
Hepatic infections (abscess, hydatid cysts)	69.57%	16
Total Respondents: 23		

Powered by SurveyMonkey

Q8: Tumor Type:

Answered: 23   Skipped: 1

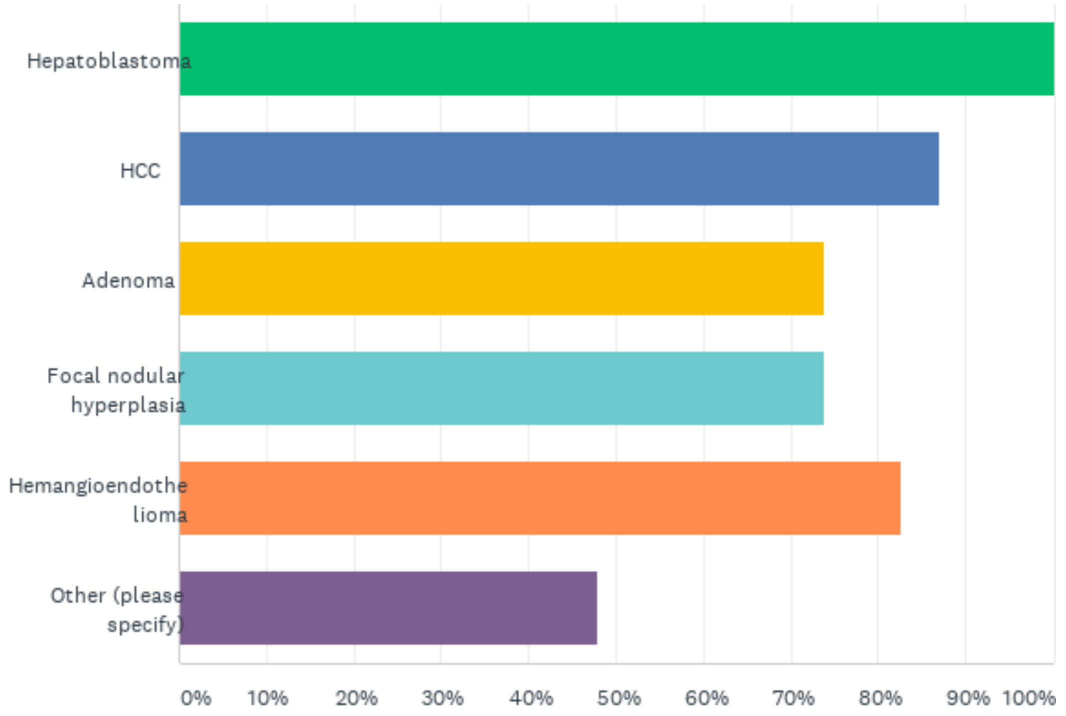

Q8: Tumor Type:

Answered: 23   Skipped: 1

ANSWER CHOICES	RESPONSES	
Hepatoblastoma	100.00%	23
HCC	86.96%	20
Adenoma	73.91%	17
Focal nodular hyperplasia	73.91%	17
Hemangioendothelioma	82.61%	19
Other (please specify)	47.83%	11
Total Respondents: 23		

Powered by SurveyMonkey

Q9: Operation performed by (multiple entries possible):

Answered: 23   Skipped: 1

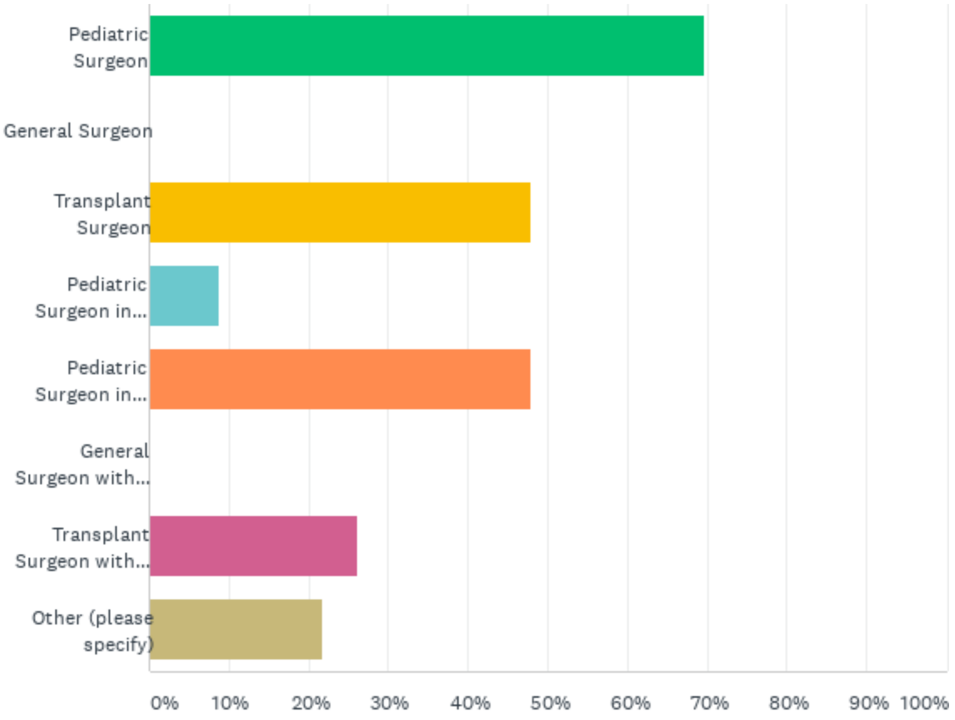

Powered by SurveyMonkey

Q9: Operation performed by (multiple entries possible):

Answered: 23   Skipped: 1

ANSWER CHOICES	RESPONSES	
Pediatric Surgeon	69.57%	16
General Surgeon	0.00%	0
Transplant Surgeon	47.83%	11
Pediatric Surgeon in cooperation with General Surgeon	8.70%	2
Pediatric Surgeon in cooperation with Transplant Surgeon	47.83%	11
General Surgeon with assistance by Pediatric Surgeon	0.00%	0
Transplant Surgeon with assistance by Pediatric Surgeon	26.09%	6
Other (please specify)	21.74%	5
Total Respondents: 23		

Q10: Type of exposition

Answered: 23   Skipped: 1

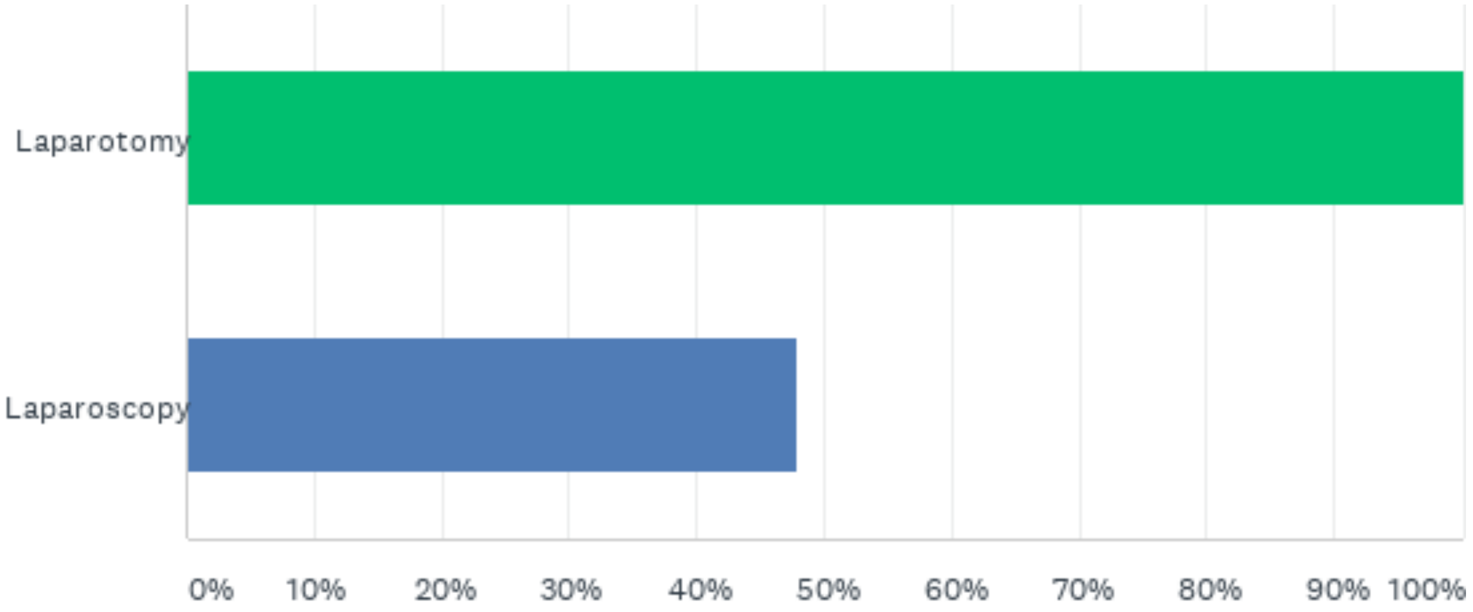

Powered by SurveyMonkey

Q10: Type of exposition

Answered: 23   Skipped: 1

ANSWER CHOICES	RESPONSES	
Laparotomy	100.00%	23
Laparoscopy	47.83%	11
Total Respondents: 23		

Q11: Type of laparotomy

Answered: 22   Skipped: 2

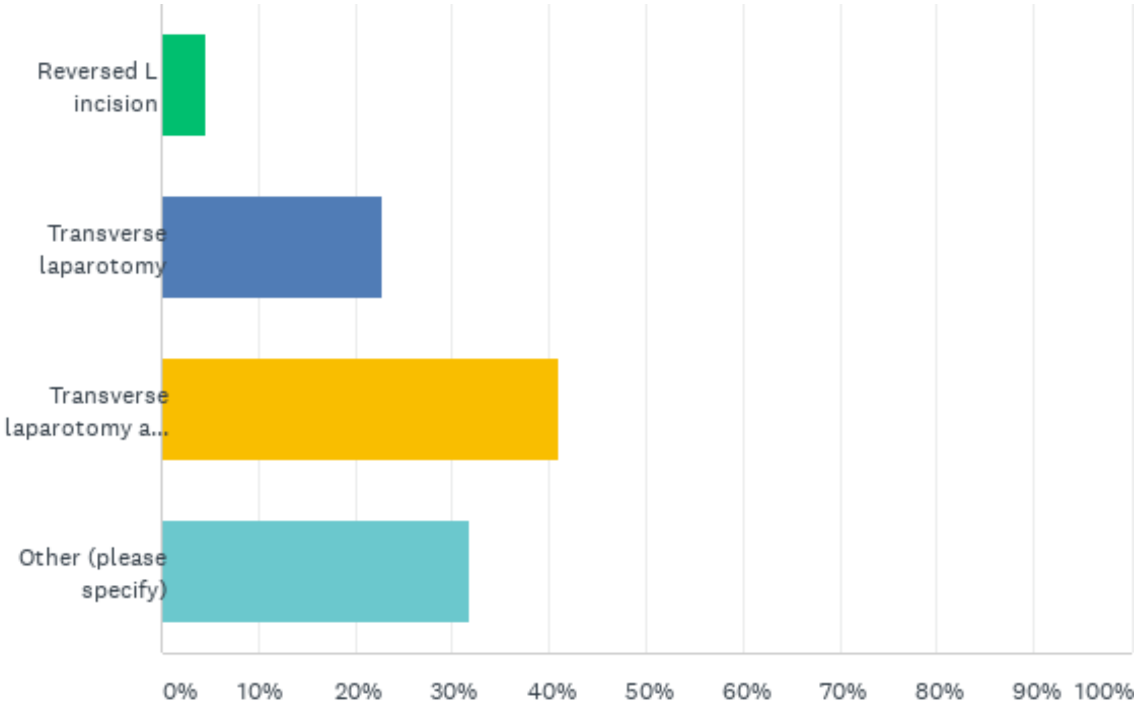

Powered by SurveyMonkey

Q11: Type of laparotomy

Answered: 22   Skipped: 2

ANSWER CHOICES	RESPONSES	
Reversed L incision	4.55%	1
Transverse laparotomy	22.73%	5
Transverse laparotomy and cranial median incision	40.91%	9
Other (please specify)	31.82%	7
TOTAL		22

Powered by SurveyMonkey

Q12: Procedures performed at your Institution:

Answered: 23   Skipped: 1

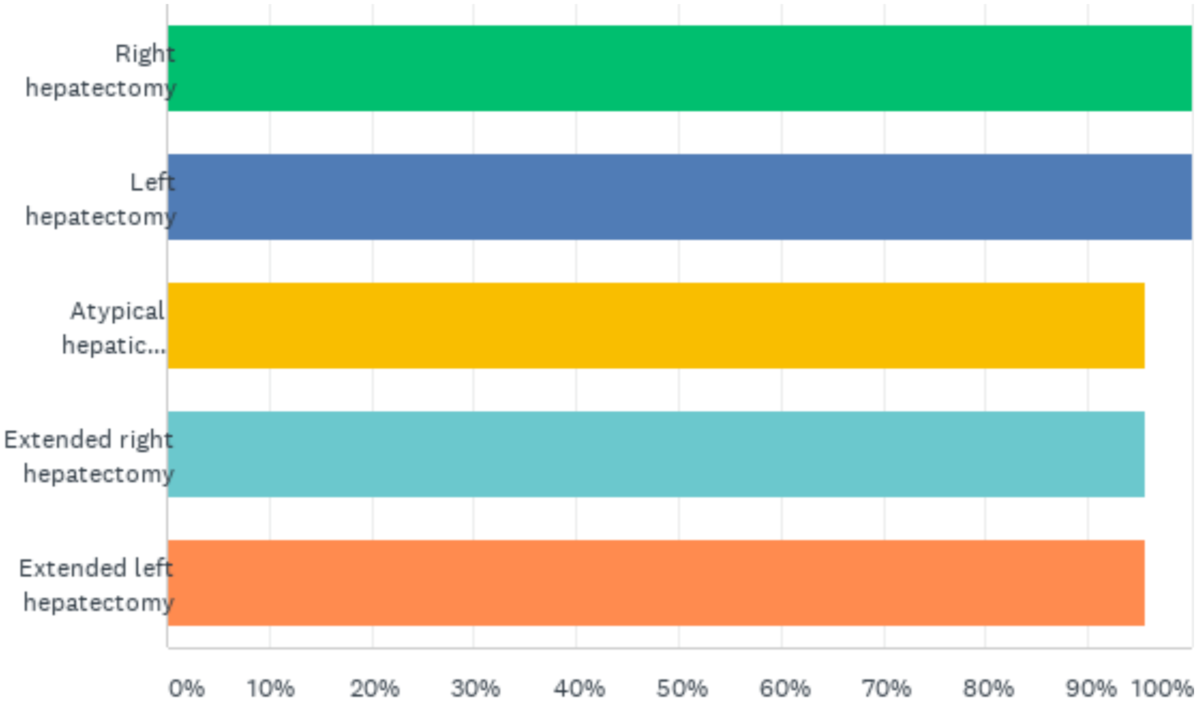

Powered by SurveyMonkey

Q12: Procedures performed at your Institution:

Answered: 23   Skipped: 1

ANSWER CHOICES	RESPONSES	
Right hepatectomy	100.00%	23
Left hepatectomy	100.00%	23
Atypical hepatic resections	95.65%	22
Extended right hepatectomy	95.65%	22
Extended left hepatectomy	95.65%	22
Total Respondents: 23		

Powered by SurveyMonkey

Q13: Vascular exclusion:

Answered: 22   Skipped: 2

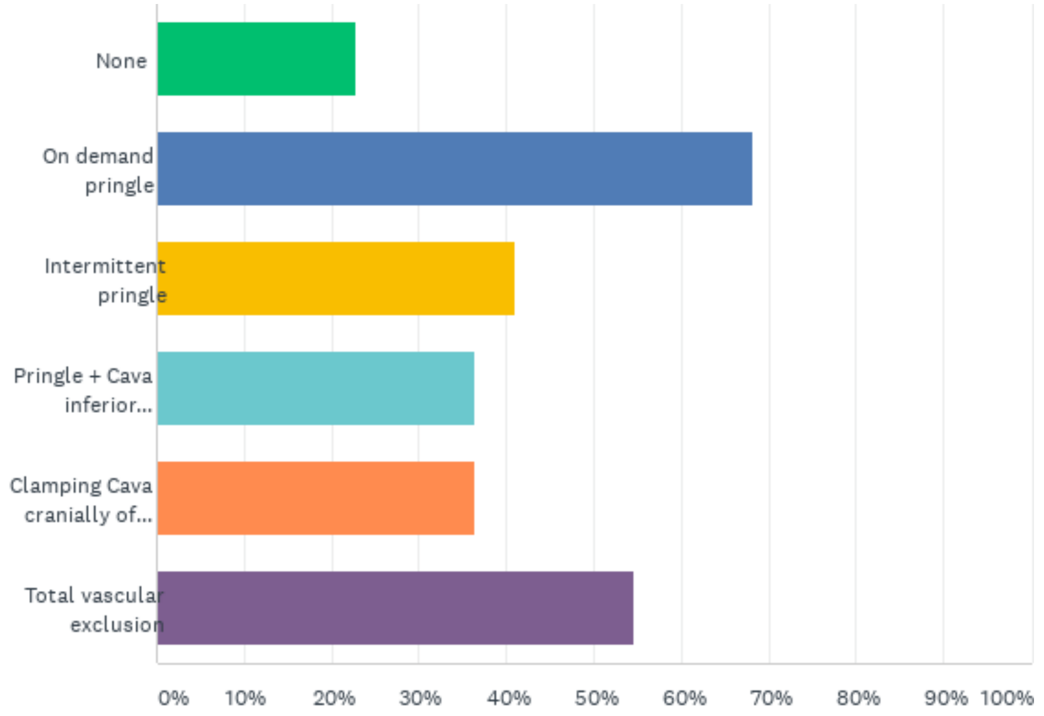

Q13: Vascular exclusion:

Answered: 22   Skipped: 2

ANSWER CHOICES	RESPONSES	
None	22.73%	5
On demand pringle	68.18%	15
Intermittent pringle	40.91%	9
Pringle + Cava inferior clamping	36.36%	8
Clamping Cava cranially of the liver	36.36%	8
Total vascular exclusion	54.55%	12
Total Respondents: 22		

Q14: Parenchymal dissection technique:

Answered: 22   Skipped: 2

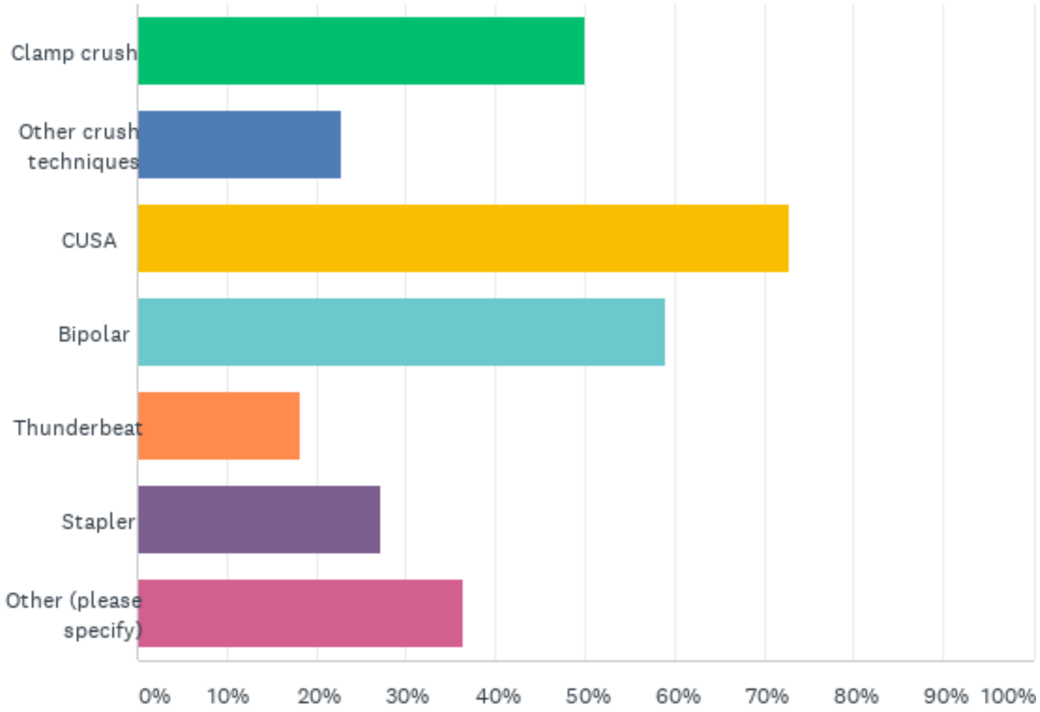

Powered by SurveyMonkey

Q14: Parenchymal dissection technique:

Answered: 22 Skipped: 2

ANSWER CHOICES	RESPONSES	
Clamp crush	50.00%	11
Other crush techniques	22.73%	5
CUSA	72.73%	16
Bipolar	59.09%	13
Thunderbeat	18.18%	4
Stapler	27.27%	6
Other (please specify)	36.36%	8
Total Respondents: 22		

Q15: Drains:

Answered: 23   Skipped: 1

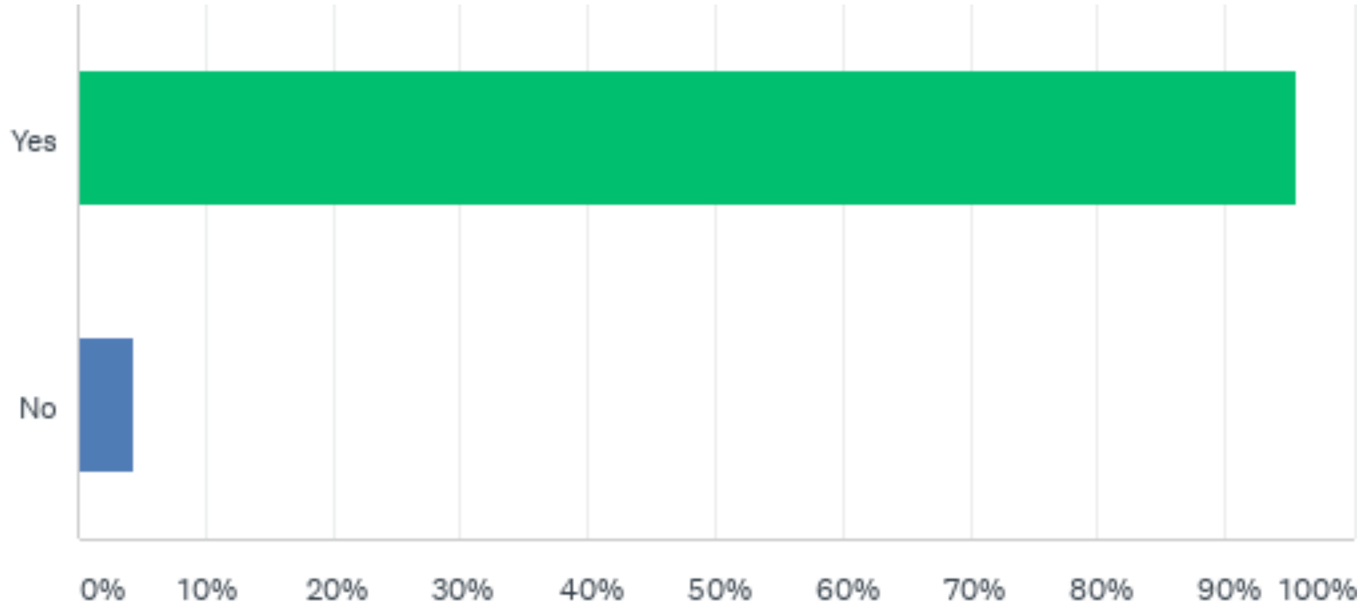

Q15: Drains:

Answered: 23   Skipped: 1

ANSWER CHOICES	RESPONSES	
Yes	95.65%	22
No	4.35%	1
TOTAL		23

Q17: Oncological Management:

Answered: 23 Skipped: 1

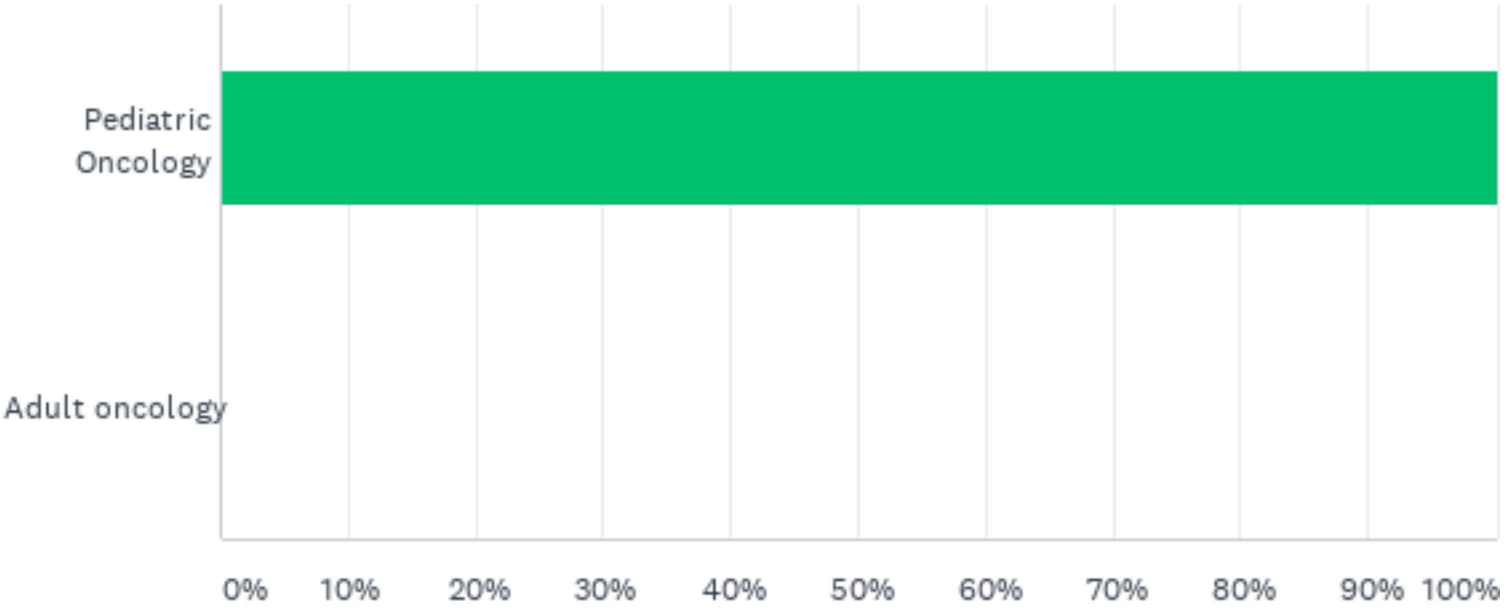

Powered by SurveyMonkey

Q17: Oncological Management:

Answered: 23   Skipped: 1

ANSWER CHOICES	RESPONSES	
Pediatric Oncology	100.00%	23
Adult oncology	0.00%	0
TOTAL		23

Q18: Decision making by:

Answered: 23   Skipped: 1

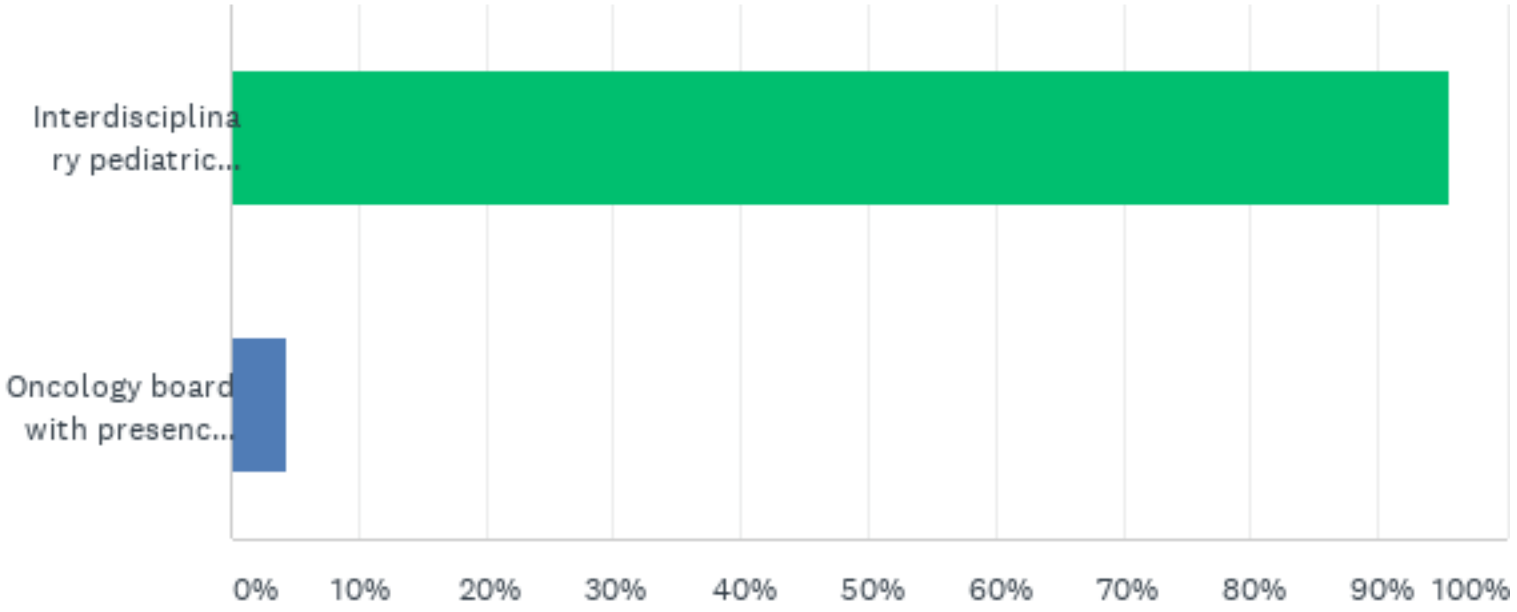

Powered by SurveyMonkey

Q18: Decision making by:

Answered: 23   Skipped: 1

ANSWER CHOICES	RESPONSES	
Interdisciplinary pediatric oncology board	95.65%	22
Oncology board with presence of General surgeons	4.35%	1
TOTAL		23

Q19: Chemotherapy performed at:

Answered: 23   Skipped: 1

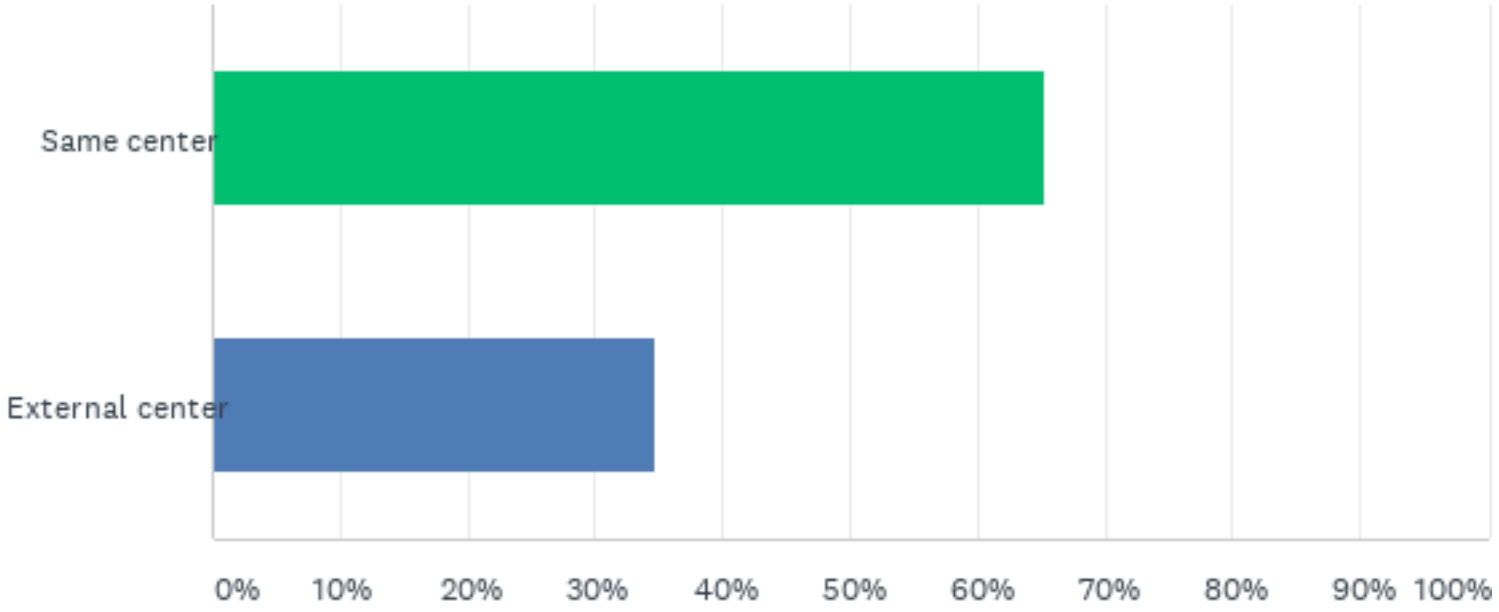

Q19: Chemotherapy performed at:

Answered: 23 Skipped: 1

ANSWER CHOICES	RESPONSES	
Same center	65.22%	15
External center	34.78%	8
TOTAL		23

Powered by SurveyMonkey

Q25: In our Center we perform pediatric liver transplantation

Answered: 23   Skipped: 1

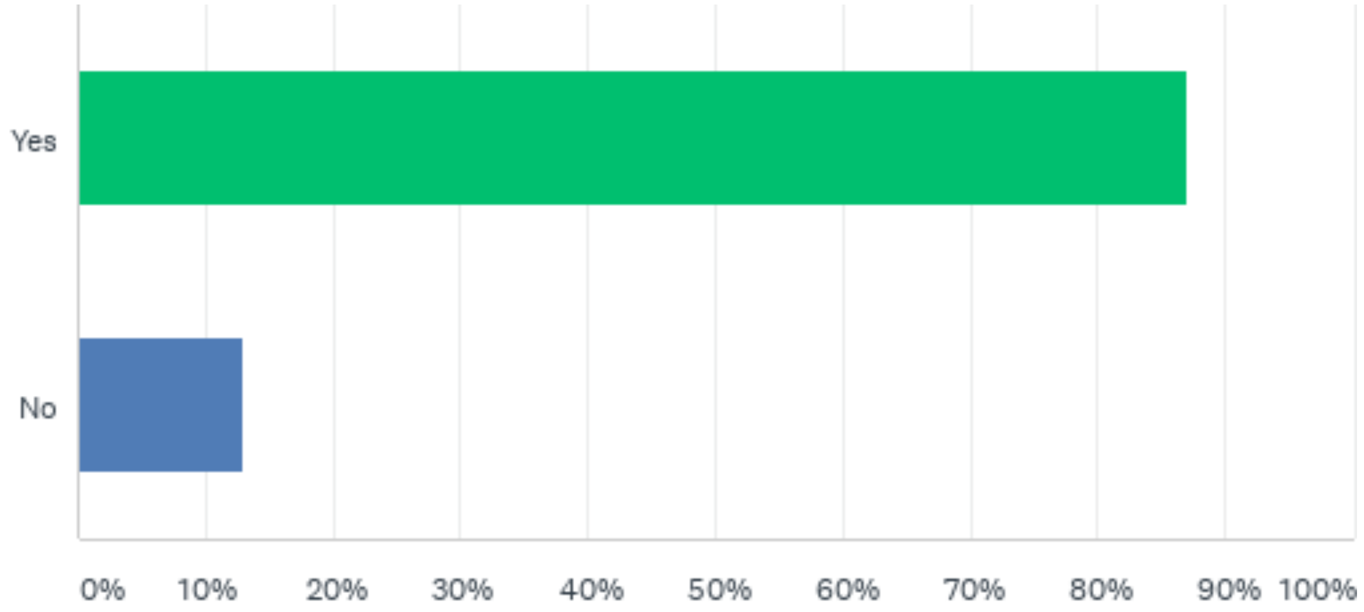

Q25: In our Center we perform pediatric liver transplantation

Answered: 23   Skipped: 1

ANSWER CHOICES	RESPONSES	
Yes	86.96%	20
No	13.04%	3
TOTAL		23

Powered by SurveyMonkey

Q26: Transplantations are performed by:

Answered: 21   Skipped: 3

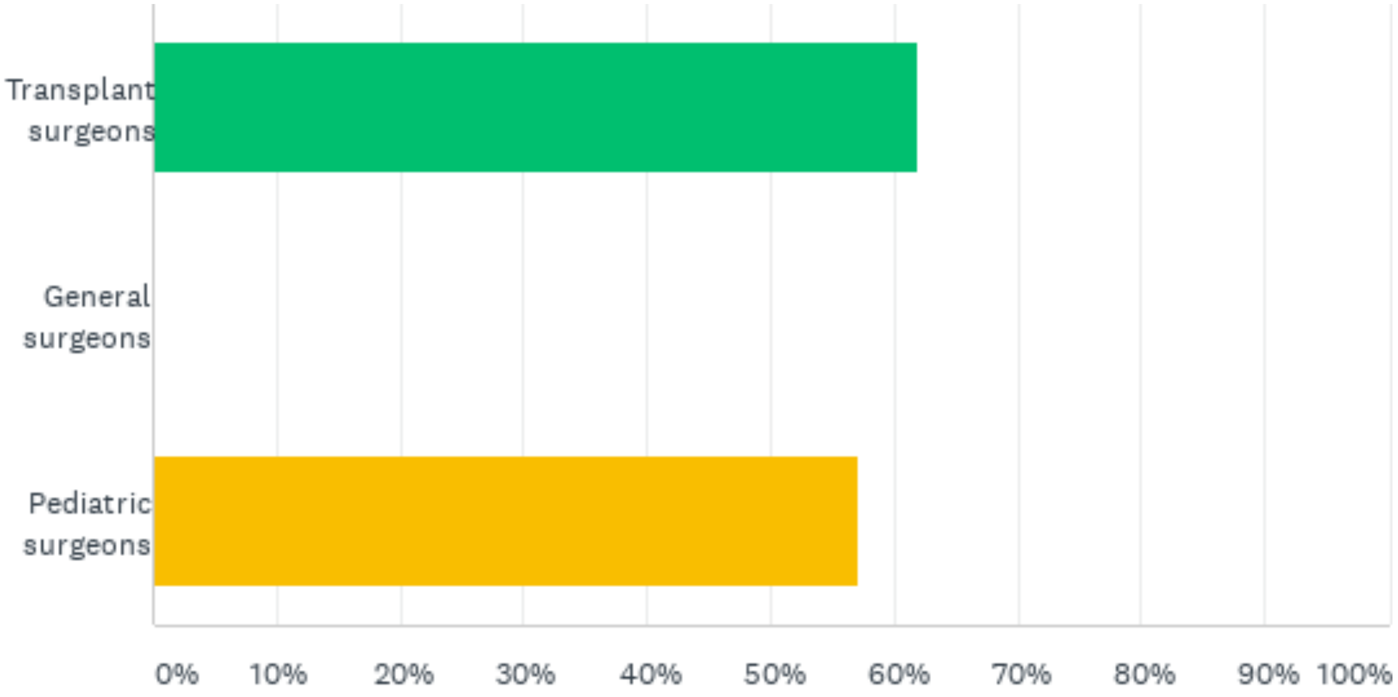

Q26: Transplantations are performed by:

Answered: 21   Skipped: 3

ANSWER CHOICES	RESPONSES	
Transplant surgeons	61.90%	13
General surgeons	0.00%	0
Pediatric surgeons	57.14%	12
Total Respondents: 21		

Powered by SurveyMonkey

Q27: Post transplant management is performed by:

Answered: 21   Skipped: 3

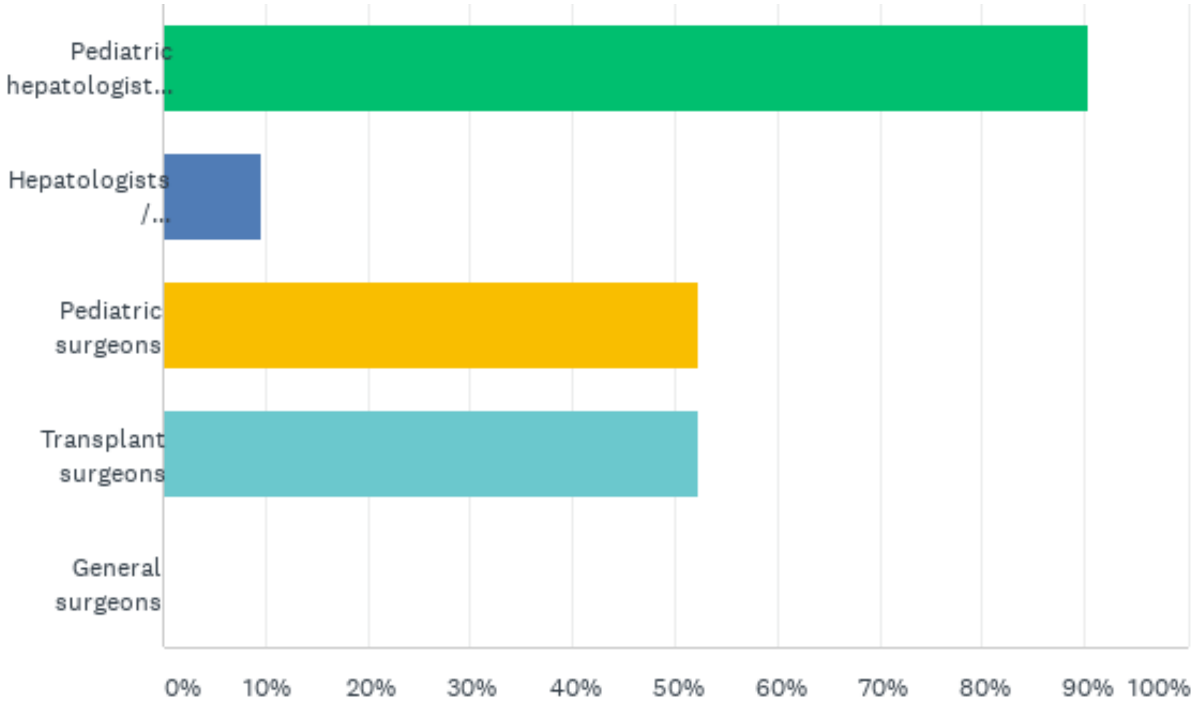

Powered by SurveyMonkey

Q27: Post transplant management is performed by:

Answered: 21   Skipped: 3

ANSWER CHOICES	RESPONSES	
Pediatric hepatologists / gastroenterologists	90.48%	19
Hepatologists / Gastroenterologists	9.52%	2
Pediatric surgeons	52.38%	11
Transplant surgeons	52.38%	11
General surgeons	0.00%	0
Total Respondents: 21		

Q28: The transplant program includes:

Answered: 20   Skipped: 4

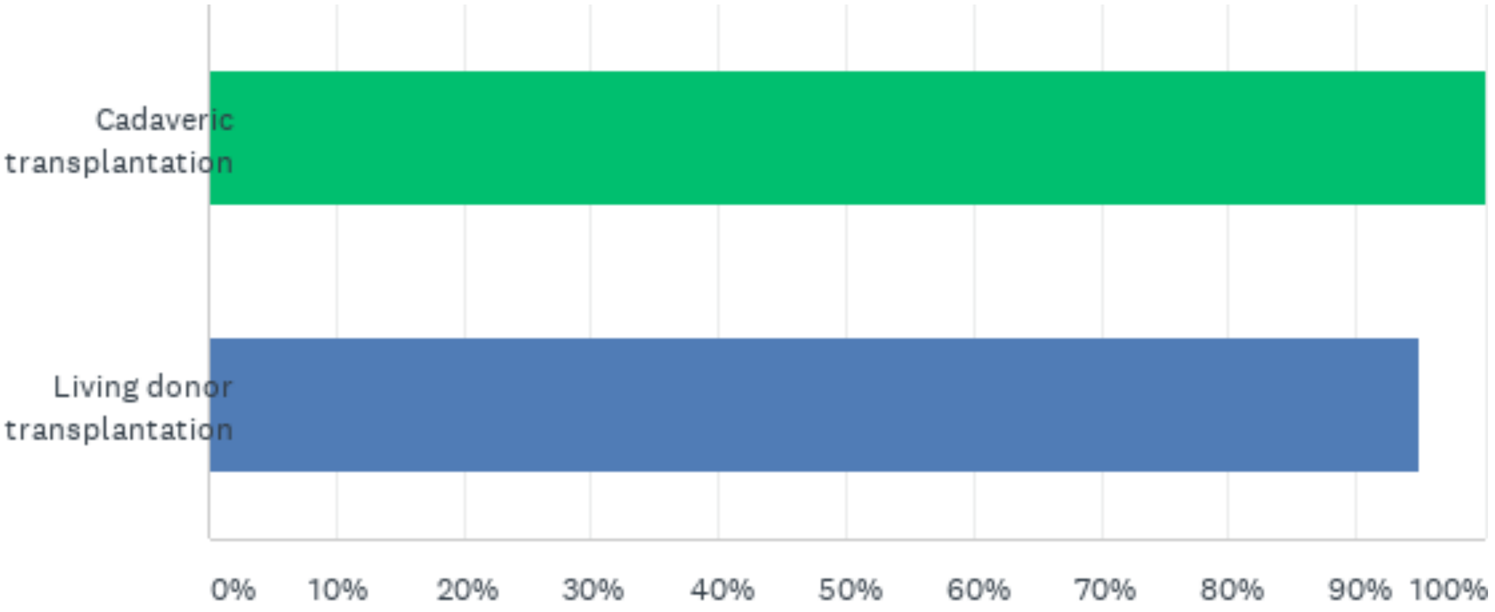

Q28: The transplant program includes:

Answered: 20   Skipped: 4

ANSWER CHOICES	RESPONSES	
Cadaveric transplantation	100.00%	20
Living donor transplantation	95.00%	19
Total Respondents: 20		

Q29: For cadaveric transplantation:

Answered: 20   Skipped: 4

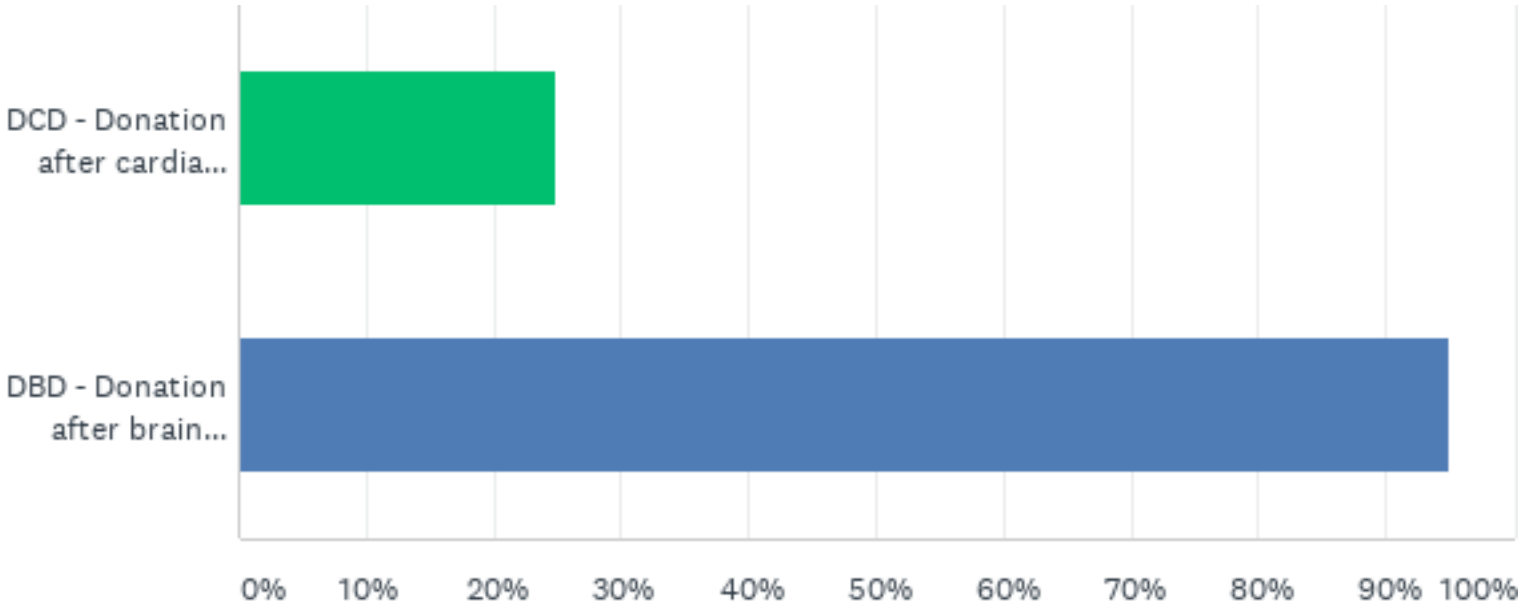

Powered by SurveyMonkey

Q29: For cadaveric transplantation:

Answered: 20   Skipped: 4

ANSWER CHOICES	RESPONSES	
DCD - Donation after cardiac death	25.00%	5
DBD - Donation after brain death	95.00%	19
Total Respondents: 20		

Q30: Pediatric liver transplantation includes:

Answered: 20 Skipped: 4

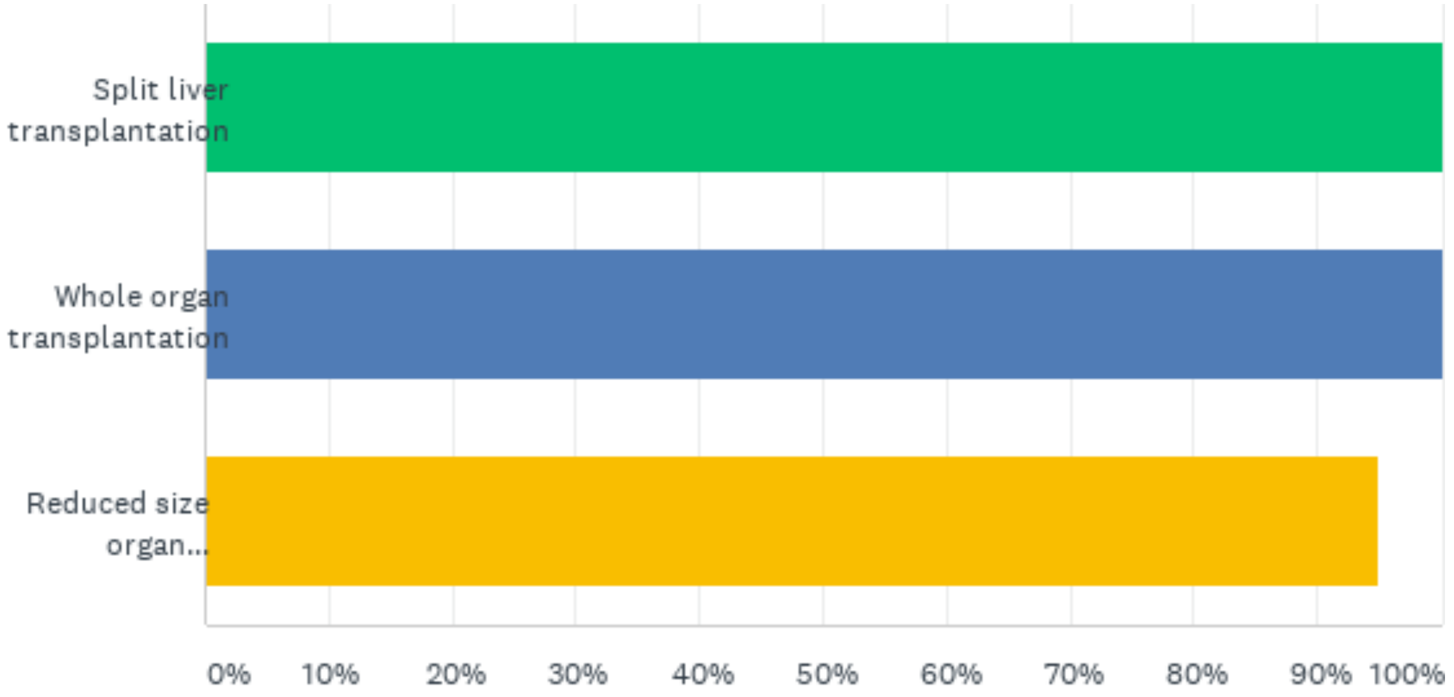

Powered by SurveyMonkey

Q30: Pediatric liver transplantation includes:

Answered: 20   Skipped: 4

ANSWER CHOICES	RESPONSES	
Split liver transplantation	100.00%	20
Whole organ transplantation	100.00%	20
Reduced size organ transplantation	95.00%	19
Total Respondents: 20		

Q31: The pediatric Transplantation program includes:

Answered: 18 Skipped: 6

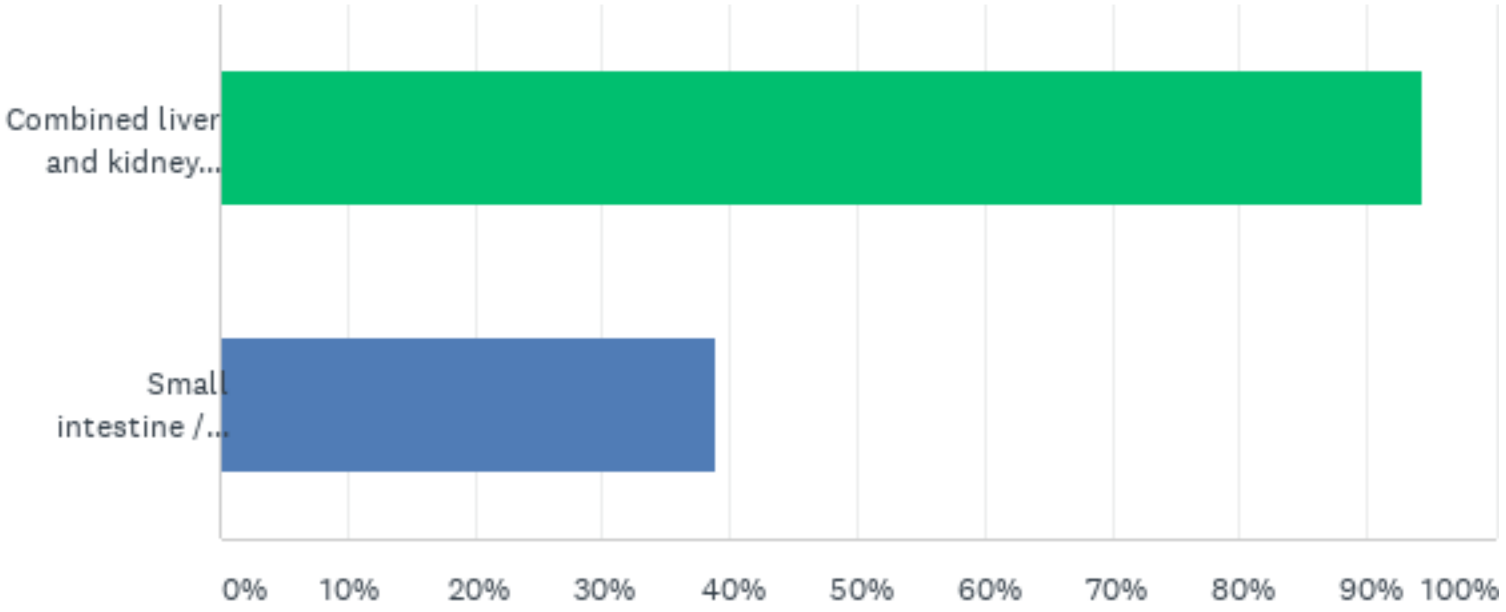

Powered by SurveyMonkey

Q31: The pediatric Transplantation program includes:

Answered: 18   Skipped: 6

ANSWER CHOICES	RESPONSES	
Combined liver and kidney transplantation	94.44%	17
Small intestine / multivisceral transplantation	38.89%	7
Total Respondents: 18		

Powered by SurveyMonkey

Delphi Questionnaire - Pediatric Liver Surgery and Transplantation

Tuesday, June 29, 2021

Powered by SurveyMonkey

Powered by SurveyMonkey

9
Total Responses

Date Created: Friday, March 05, 2021

Complete Responses: 9

Powered by SurveyMonkey

Q1: Centers performing pediatric liver surgery should contain all necessary facilities for diagnostics (Sonography, CT, MRI)

Answered: 9   Skipped: 0

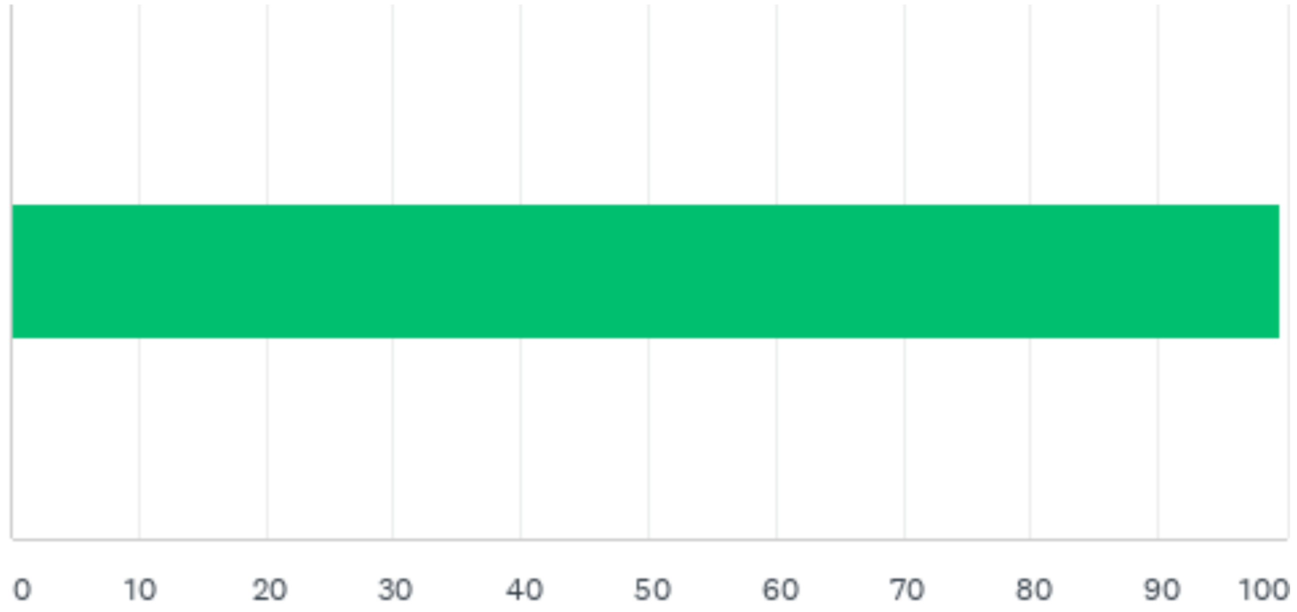

Powered by SurveyMonkey

Q1: Centers performing pediatric liver surgery should contain all necessary facilities for diagnostics (Sonography, CT, MRI)

Answered: 9   Skipped: 0

ANSWER CHOICES	AVERAGE NUMBER	TOTAL NUMBER	RESPONSES
	100	896	9
Total Respondents: 9			

Powered by SurveyMonkey

Q2: Centers performing pediatric liver surgery should contain all options for radiological interventions during diagnostics (biopsies)

Answered: 9 Skipped: 0

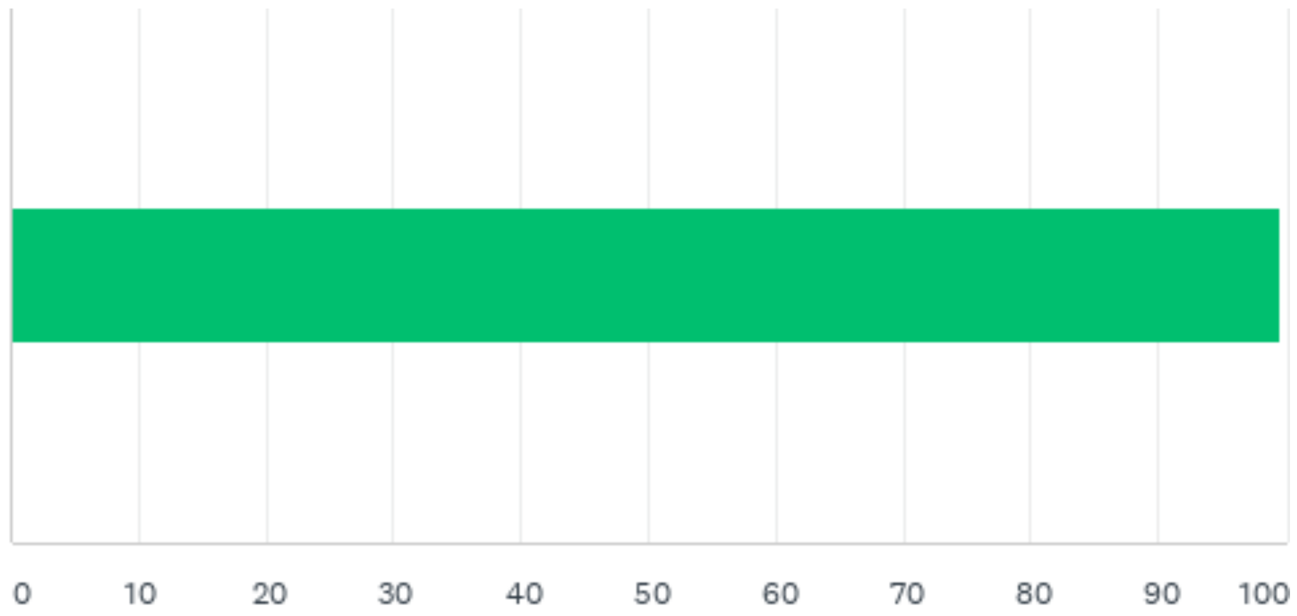

Q2: Centers performing pediatric liver surgery should contain all options for radiological interventions during diagnostics (biopsies)

Answered: 9   Skipped: 0

ANSWER CHOICES	AVERAGE NUMBER	TOTAL NUMBER	RESPONSES
	100	896	9
Total Respondents: 9			

Q3: Centers performing pediatric liver surgery need the facilities for preoperative radiological interventions (portal vein embolization)

Answered: 9   Skipped: 0

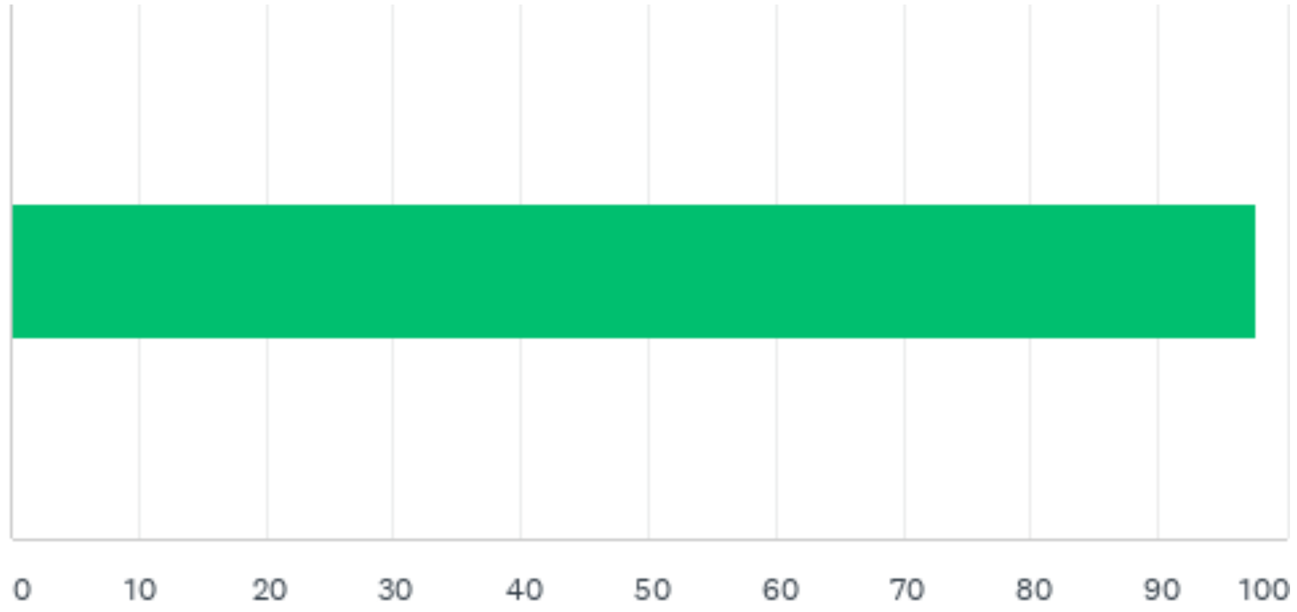

Powered by SurveyMonkey

Q3: Centers performing pediatric liver surgery need the facilities for preoperative radiological interventions (portal vein embolization)

Answered: 9   Skipped: 0

ANSWER CHOICES	AVERAGE NUMBER	TOTAL NUMBER	RESPONSES
	98	879	9
Total Respondents: 9			

Powered by SurveyMonkey

Q4: Centers performing pediatric liver surgery should contain the option for pre- and postoperative pediatric ERCP (with interventions)

Answered: 9   Skipped: 0

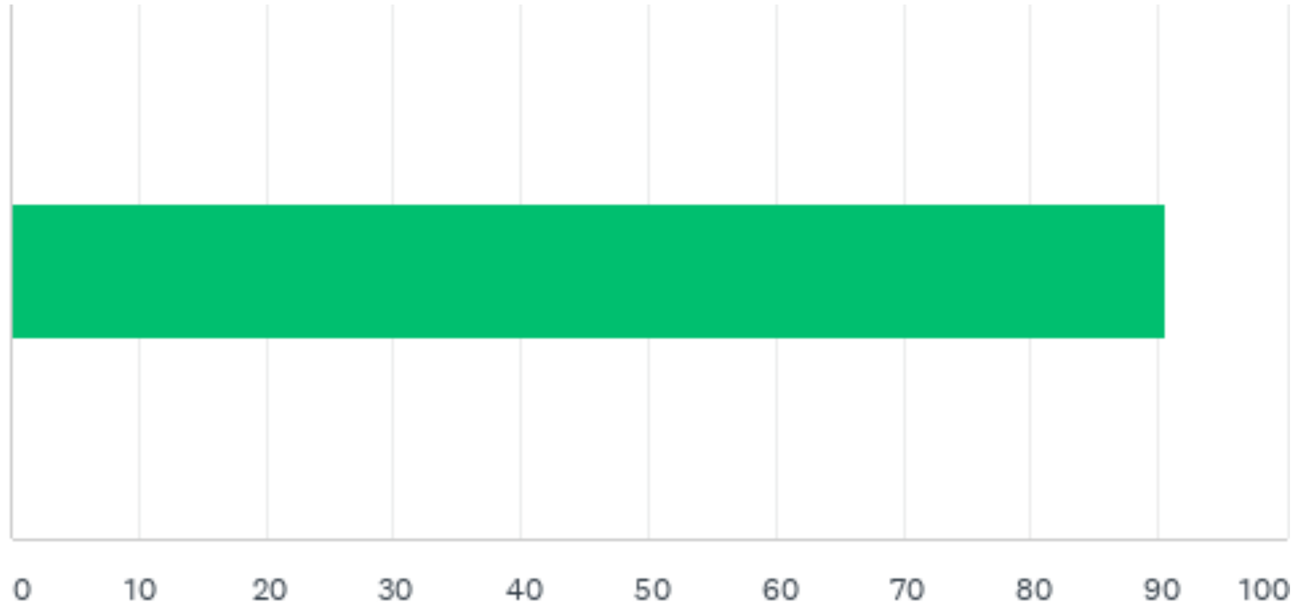

Powered by SurveyMonkey

Q4: Centers performing pediatric liver surgery should contain the option for pre- and postoperative pediatric ERCP (with interventions)

Answered: 9   Skipped: 0

ANSWER CHOICES	AVERAGE NUMBER	TOTAL NUMBER	RESPONSES
	91	815	9
Total Respondents: 9			

Powered by SurveyMonkey

Q5: Centers performing pediatric liver surgery should contain the option for pre- and postoperative PTC (with interventions)

Answered: 9   Skipped: 0

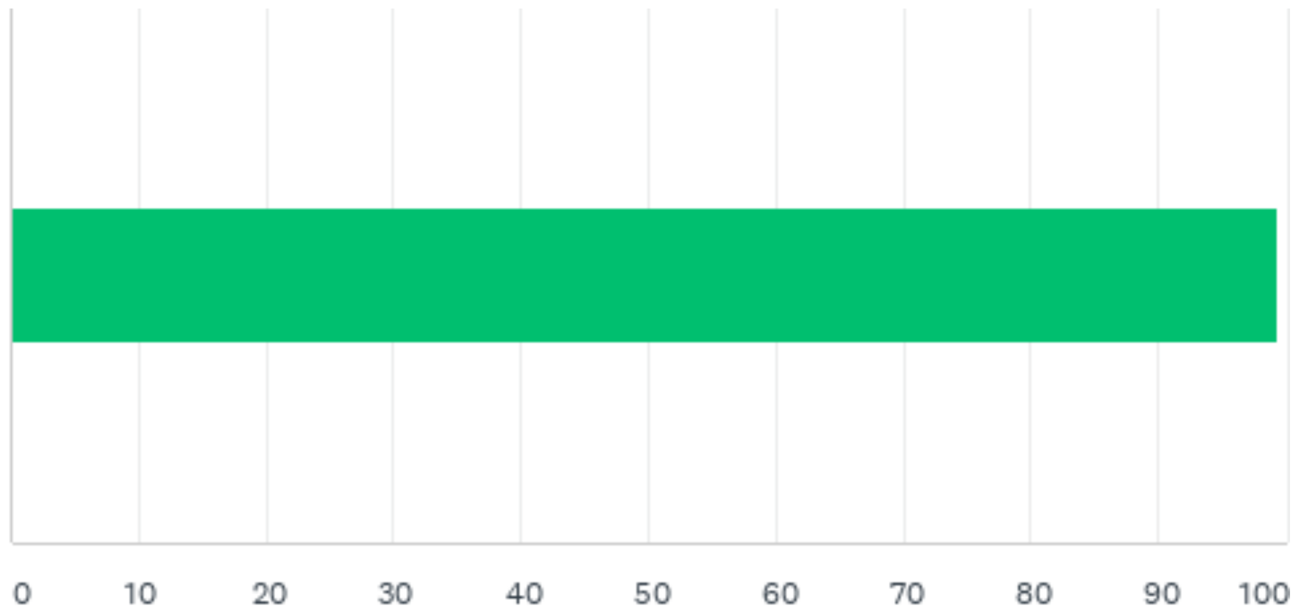

Q5: Centers performing pediatric liver surgery should contain the option for pre- and postoperative PTC (with interventions)

Answered: 9   Skipped: 0

ANSWER CHOICES	AVERAGE NUMBER	TOTAL NUMBER	RESPONSES
	99	895	9
Total Respondents: 9			

Powered by SurveyMonkey

Q6: Pediatric liver surgery should rather be performed by transplant surgeons then by pediatric surgeons

Answered: 9   Skipped: 0

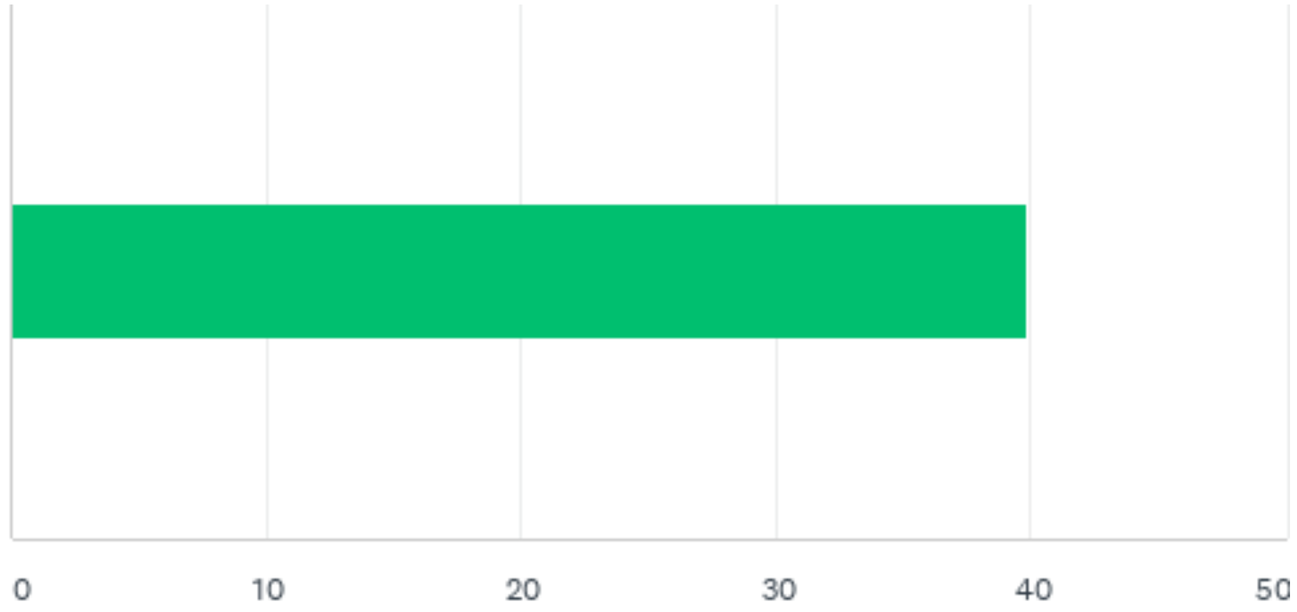

Powered by SurveyMonkey

Q6: Pediatric liver surgery should rather be performed by transplant surgeons then by pediatric surgeons

Answered: 9   Skipped: 0

ANSWER CHOICES	AVERAGE NUMBER	TOTAL NUMBER	RESPONSES
	40	359	9
Total Respondents: 9			

Powered by SurveyMonkey

Q7: It is necessary that the surgical team for pediatric liver tumors include a pediatric surgeon and a transplant surgeon

Answered: 9   Skipped: 0

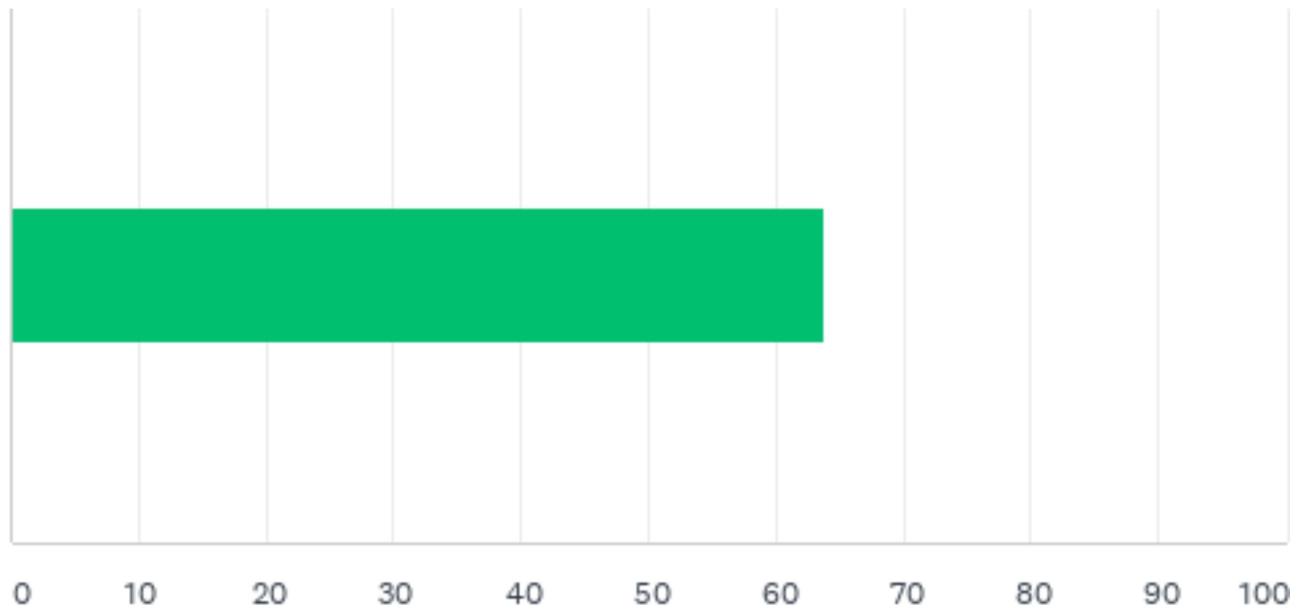

Q7: It is necessary that the surgical team for pediatric liver tumors include a pediatric surgeon and a transplant surgeon

Answered: 9   Skipped: 0

ANSWER CHOICES	AVERAGE NUMBER	TOTAL NUMBER	RESPONSES
	64	574	9
Total Respondents: 9			

Q8: Pediatric liver surgery should be performed via laparotomy

Answered: 9 Skipped: 0

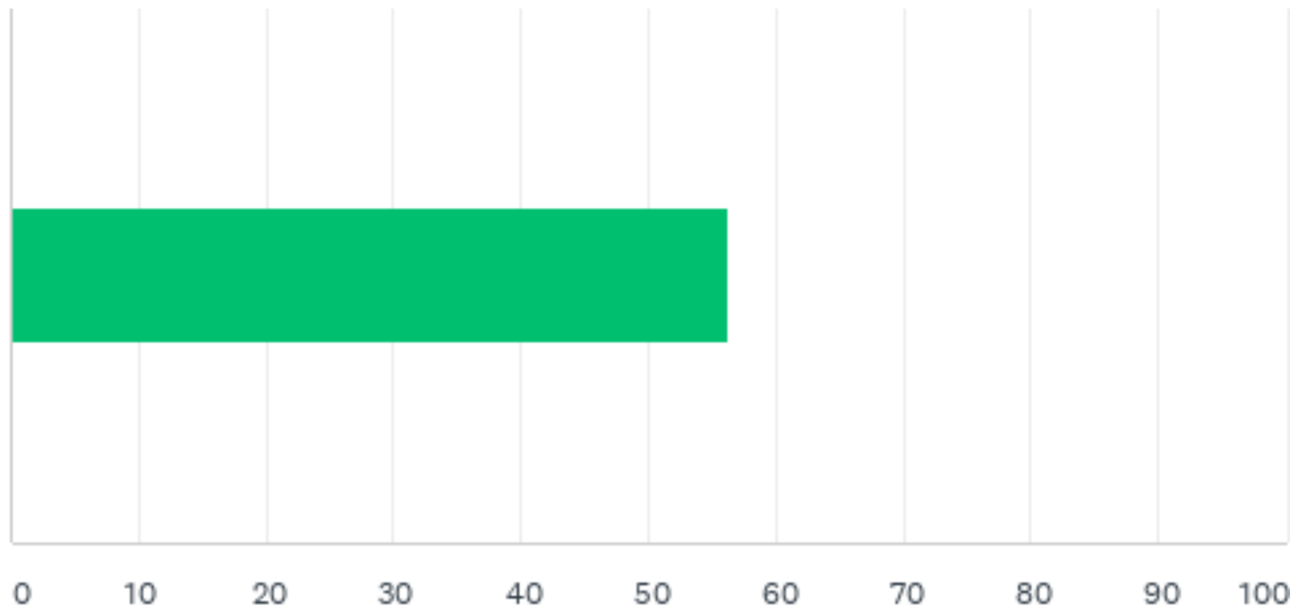

Powered by SurveyMonkey

Q8: Pediatric liver surgery should be performed via laparotomy

Answered: 9   Skipped: 0

ANSWER CHOICES	AVERAGE NUMBER	TOTAL NUMBER	RESPONSES
	56	507	9
Total Respondents: 9			

Q9: Laparoscopy should be an option for minor and major cases of pediatric liver surgery

Answered: 9   Skipped: 0

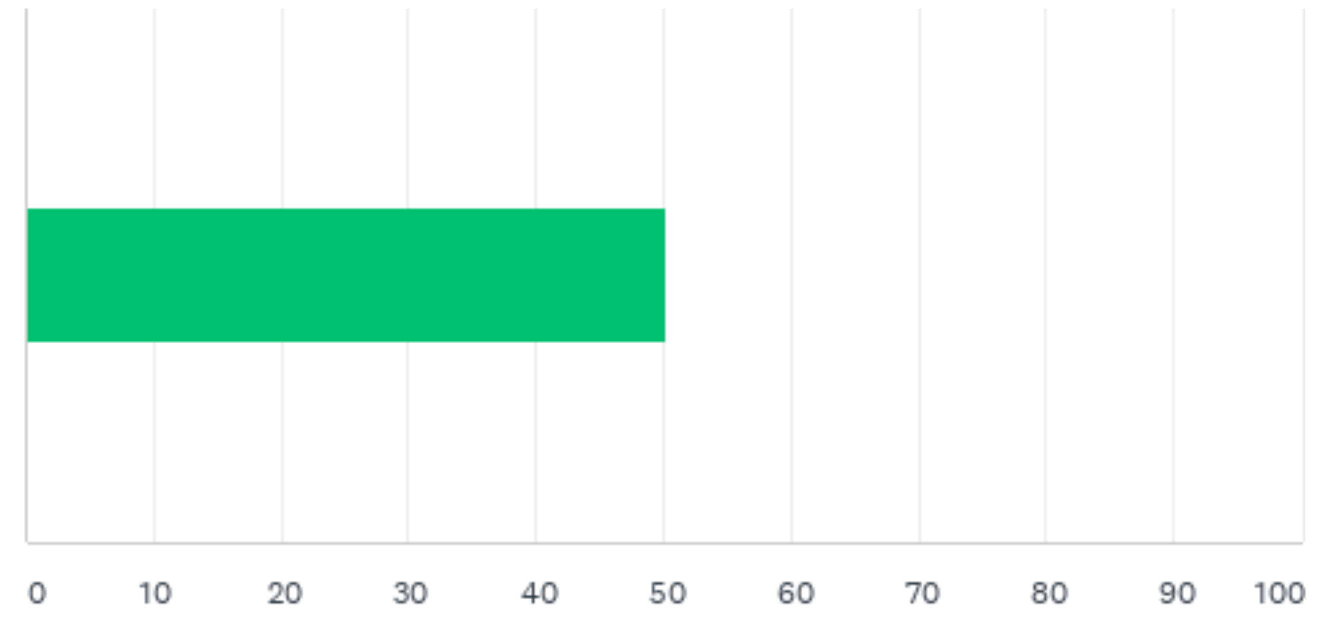

Powered by SurveyMonkey

Q9: Laparoscopy should be an option for minor and major cases of pediatric liver surgery

Answered: 9   Skipped: 0

ANSWER CHOICES	AVERAGE NUMBER	TOTAL NUMBER	RESPONSES
	50	451	9
Total Respondents: 9			

Q10: The knowledge of techniques for different types of vascular exclusion is mandatory for pediatric liver surgery

Answered: 9   Skipped: 0

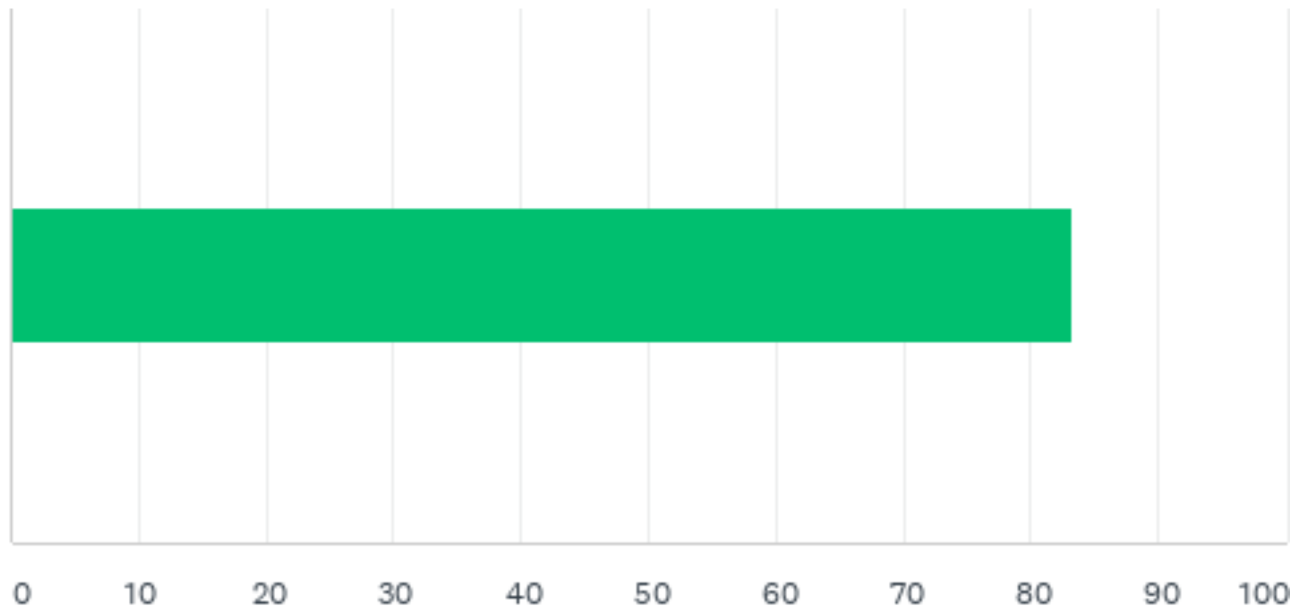

Powered by SurveyMonkey

Q10: The knowledge of techniques for different types of vascular exclusion is mandatory for pediatric liver surgery

Answered: 9   Skipped: 0

ANSWER CHOICES	AVERAGE NUMBER	TOTAL NUMBER	RESPONSES
	83	749	9
Total Respondents: 9			

Powered by SurveyMonkey

Q11: Pringle is necessary during liver parenchymal dissection

Answered: 8   Skipped: 1

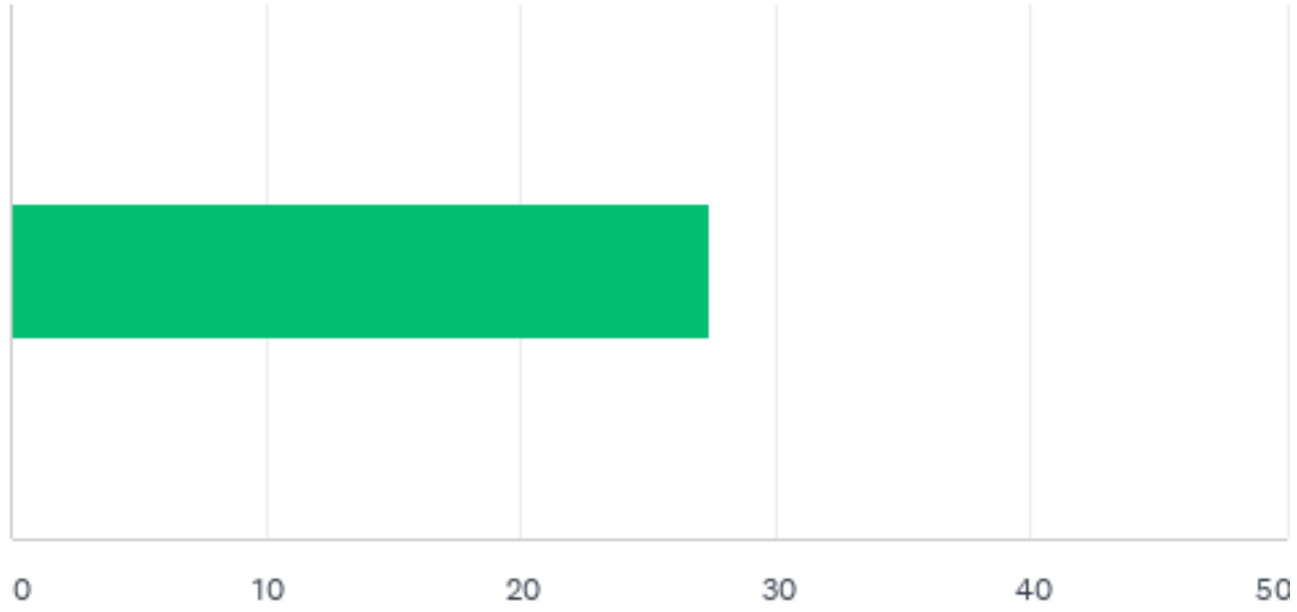

Q11: Pringle is necessary during liver parenchymal dissection

Answered: 8 Skipped: 1

ANSWER CHOICES	AVERAGE NUMBER	TOTAL NUMBER	RESPONSES
	27	219	8
Total Respondents: 8			

Q12: Total vascular exclusion should be prepared during major liver resection

Answered: 8   Skipped: 1

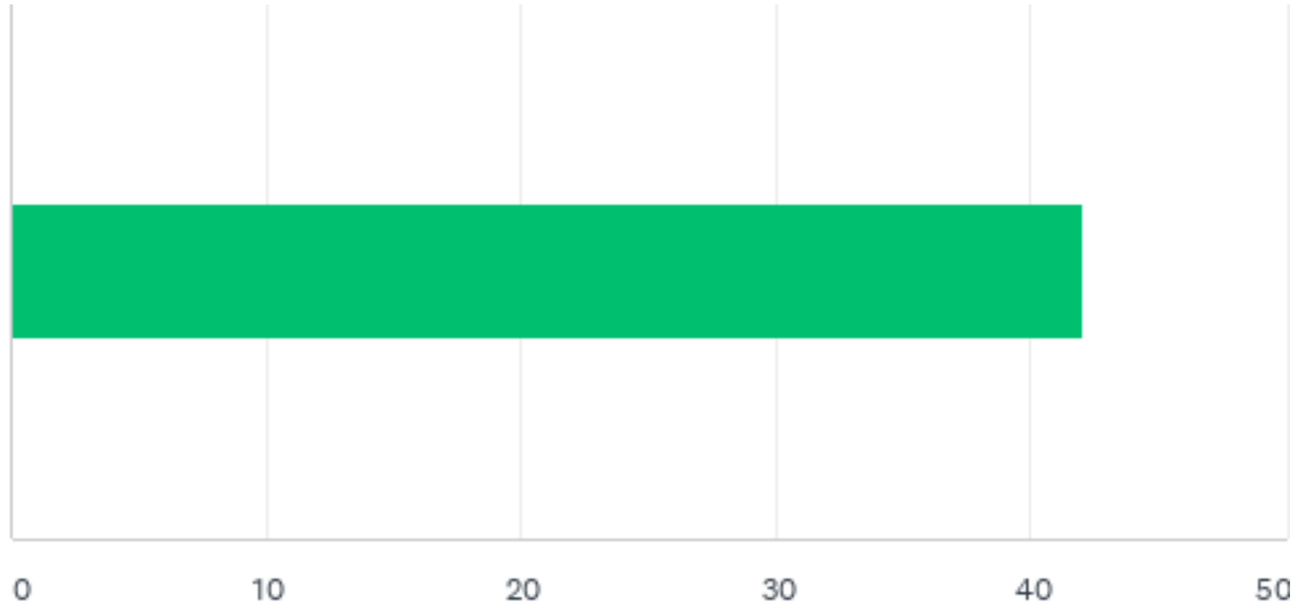

Q12: Total vascular exclusion should be prepared during major liver resection

Answered: 8   Skipped: 1

ANSWER CHOICES	AVERAGE NUMBER	TOTAL NUMBER	RESPONSES
	42	336	8
Total Respondents: 8			

Powered by SurveyMonkey

Q13: Novel equipment (CUSA, Thunderbeat) should be available in centers of pediatric liver surgery

Answered: 8   Skipped: 1

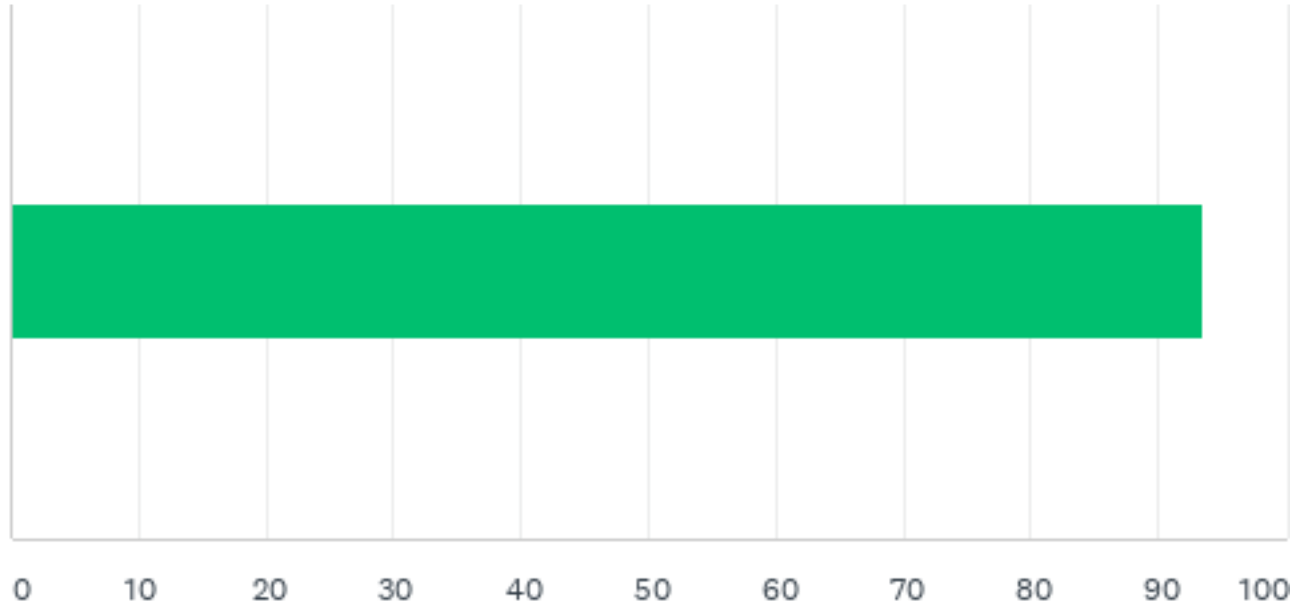

Q13: Novel equipment (CUSA, Thunderbeat) should be available in centers of pediatric liver surgery

Answered: 8 Skipped: 1

ANSWER CHOICES	AVERAGE NUMBER	TOTAL NUMBER	RESPONSES
	94	748	8
Total Respondents: 8			

Powered by SurveyMonkey

Q14: Centers treating pediatric liver tumors should include a pediatric transplant program

Answered: 8   Skipped: 1

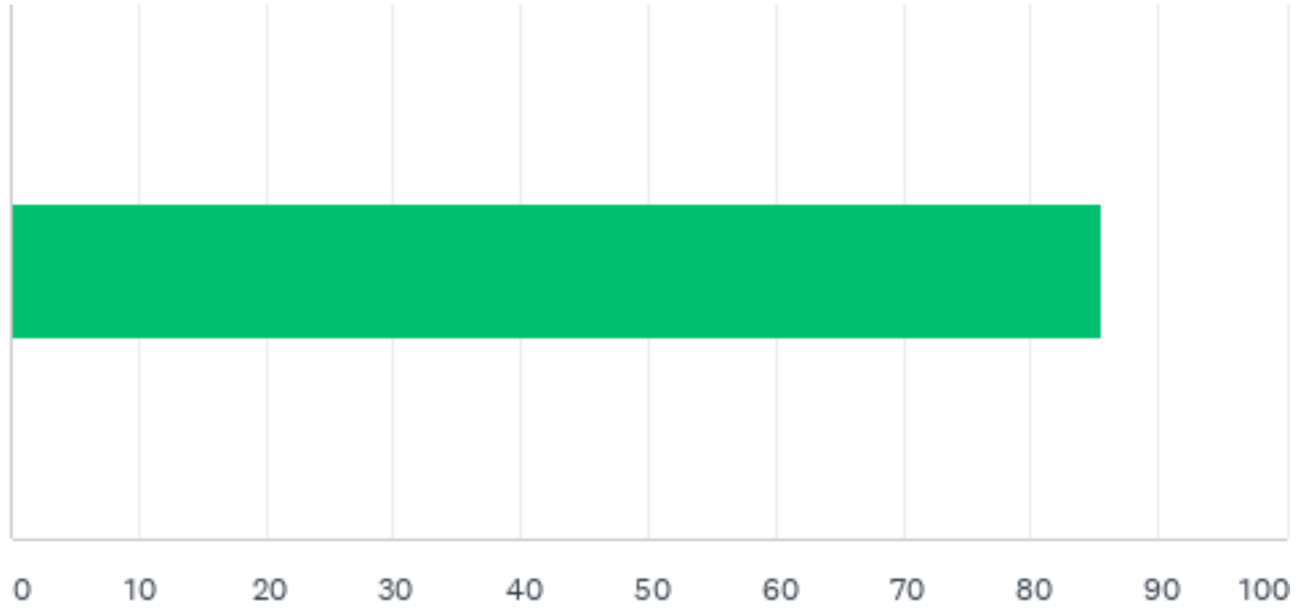

Powered by SurveyMonkey

Q14: Centers treating pediatric liver tumors should include a pediatric transplant program

Answered: 8 Skipped: 1

ANSWER CHOICES	AVERAGE NUMBER	TOTAL NUMBER	RESPONSES
	86	685	8
Total Respondents: 8			

Q15: Living donor transplantation should be an option in centers treating pediatric liver tumors

Answered: 9   Skipped: 0

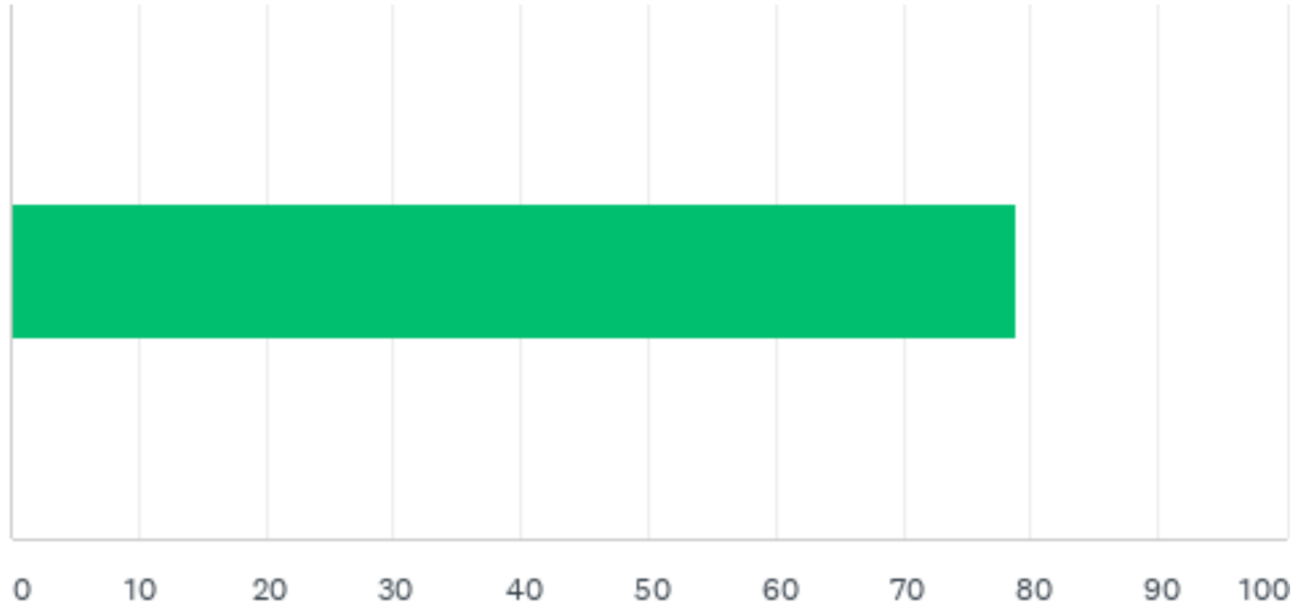

Powered by SurveyMonkey

Q15: Living donor transplantation should be an option in centers treating pediatric liver tumors

Answered: 9   Skipped: 0

ANSWER CHOICES	AVERAGE NUMBER	TOTAL NUMBER	RESPONSES
	79	710	9
Total Respondents: 9			

Q16: Pediatric liver transplantation should be performed by transplant surgeons rather than by specialized pediatric surgeons

Answered: 9   Skipped: 0

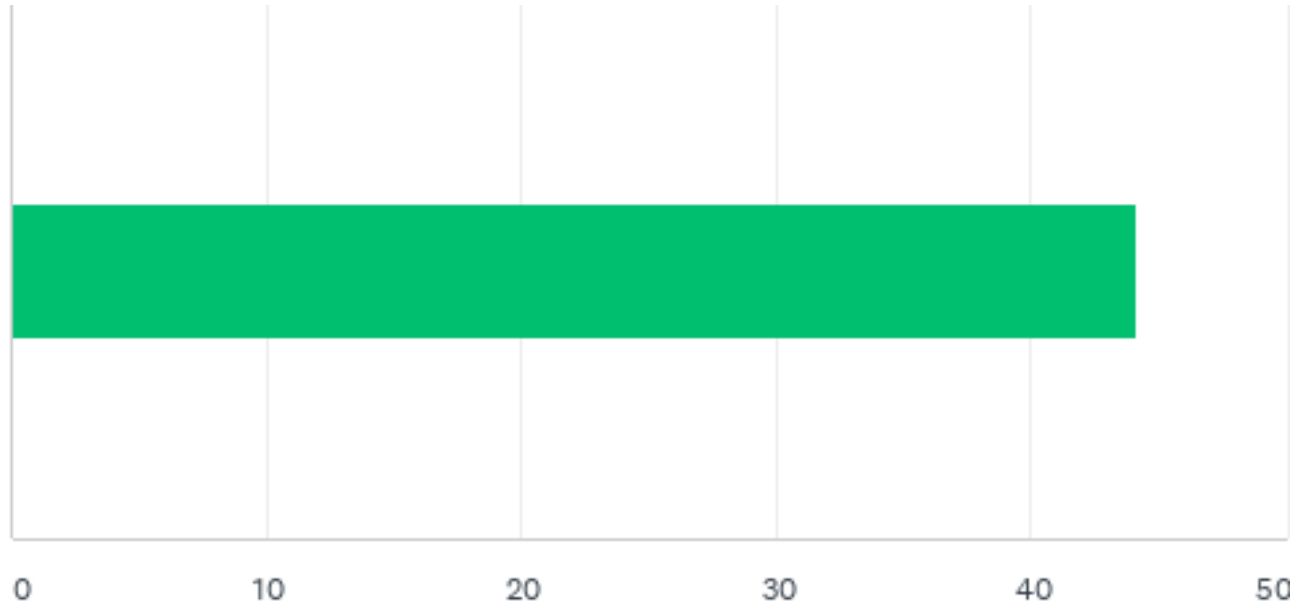

Powered by SurveyMonkey

Q16: Pediatric liver transplantation should be performed by transplant surgeons rather than by specialized pediatric surgeons

Answered: 9   Skipped: 0

ANSWER CHOICES	AVERAGE NUMBER	TOTAL NUMBER	RESPONSES
	44	397	9
Total Respondents: 9			

Powered by SurveyMonkey
